# Postfunctionalization of PAN Membranes via UV-Grafting of Charged and Zwitterionic Polymer Brushes

**DOI:** 10.1021/acs.langmuir.5c05068

**Published:** 2025-12-22

**Authors:** Timo Friedrich, Donovan Timm, Sarah Glass, Erik S. Schneider, Volkan Filiz, Wolfgang Maison

**Affiliations:** † 14915 Universität Hamburg, Department of Chemistry, Bundesstrasse 45, 20146 Hamburg, Germany; ‡ Institute of Membrane Research, Helmholtz-Zentrum Hereon, Max-Planck-Str. 1, 21502 Geesthacht, Germany

## Abstract

Polyacrylonitrile (PAN) membranes are widely used for water purification, but their susceptibility to fouling limits efficiency and lifespan. In this study, a sustainable and efficient UV-grafting process was employed to modify PAN membranes. Cationic and zwitterionic vinylbenzene- and methacrylate-based monomers, including ammonium, ammonium alcohol, *N*-oxide, carboxybetaine, sulfobetaine, and phosphobetaine groups, were used to graft polymers from the membrane surface using photoinitiators such as phenylbis­(2,4,6-trimethylbenzoyl)­phosphine oxide (BAPO) and lithium phenyl-2,4,6-trimethylbenzoylphosphinate (LAP). The modified membranes were characterized using FTIR, SEM-EDX, AFM, ζ potential analysis, permeability testing, molecular weight cutoff measurements, dye adsorption assays, porosity, and pore size analysis. Microbiological evaluation following a modified ASTM E2149-20 protocol revealed antibacterial properties for some of the grafted polymers. These findings demonstrate the potential of postfunctionalized PAN membranes for the adsorption of dyes and to mitigate fouling, both are important factors for water treatment.

## Graphical abstract

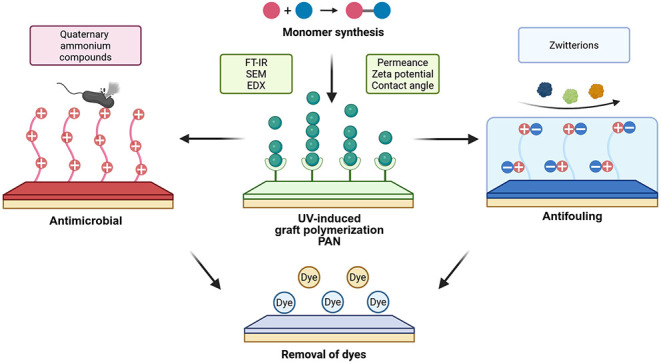

## Introduction

Access to clean drinking water remains a global challenge due to contamination with industrial effluents, pharmaceutical residues, and microbial pathogens.^1,2^ Membrane-based filtration technologies play an important role in this context. Polyacrylonitrile (PAN) membranes, for example, are widely employed in ultrafiltration, nanofiltration and reverse osmosis.^3–6^ PAN membranes are also used for separating oil from water, removing heavy metals, and in hemodialysis.^7–9^ PAN membranes are typically fabricated using electrospinning, which enables precise control over fiber diameter, and the nonsolvent-induced phase separation (NIPS) technique, which regulates membrane porosity and surface properties.^10,11^

A major limitation of PAN membranes is their susceptibility to fouling, which reduces filtration efficiency and membrane lifespan. Addressing this issue is critical for improving the sustainability and performance of PAN-based filtration systems. Membrane biofouling is primarily caused by the adhesion of proteins and bacteria, which results in a decline in membrane permeability and necessitates frequent cleaning procedures.^12^ Traditional cleaning approaches often involve chemical agents like sodium hypochlorite or hydrogen peroxide that contribute to operational costs and environmental concerns.^13^ Various strategies have been explored to modify PAN membranes to reduce fouling and enhance performance.^14^ A common approach is the introduction of hydrophilic functional groups via chemical modification, plasma treatment, surface grafting, blending or composites.^15–19^ A widely studied approach is the incorporation of poly­(ethylene glycol) (PEG), which is known for its hydrophilicity and nonadhesive properties. PEG immobilization on PAN membranes has been shown to significantly reduce protein adhesion, improve membrane wettability, and enhance flux recovery after filtration. PEG-functionalized membranes can achieve a 6-fold increase in bovine serum albumin (BSA) solution flux while reducing total fouling and protein adsorption by more than 60% compared to unfunctionalized membranes.^20,21^

Zwitterionic and cationic modifications are promising strategies for improving nonadhesive and antimicrobial material properties.^22–24^ Zwitterions form a hydration layer that prevents protein adsorption and bacterial adhesion.^25^ Cationic modifications, on the other hand, can lead to antimicrobial properties by contact-active disruption of bacterial cell membranes.^26,27^ PAN membranes functionalized with sulfobetaine or carboxybetaine groups have shown excellent resistance to protein adsorption and improved antifouling performance.^28–31^ In some cases, the reversible adsorption and desorption of proteins was controlled by adjusting salt concentrations.^32,33^ In addition, PAN membranes modified with zwitterionic coatings are efficient for oil/water separation while maintaining excellent water permeability and long-term stability.^34,35^ Amine-functionalized PAN membranes have been successfully applied for removing contaminants such as arsenate and chromate from water.^36,37^ These modifications not only increase anion adsorption but also improve overall membrane performance without significantly affecting permeability.

This study compares the effects of various grafted charged polymers and different polymer backbones on the properties of PAN membranes. A variety of cationic (ammonium), zwitterionic (*N*-oxide, sulfobetaine, carboxybetaine, phosphobetaine) and mixed-charge (double substituted DABCO derivatives) polymer brushes were grafted via a UV grafting technique onto PAN membranes.^38,39^ For the design of zwitterionic PAN membranes, tertiary amine-based building blocks were chosen due to their low cost, commercial availability, and suitability as precursors for carboxybetaine, sulfobetaine, and *N*-oxide structures. Membranes bearing phosphobetaine groups were prepared by graft-polymerization of commercially available monomers. Reagents for postfunctionalization of graft polymers were selected to generate short spacers between the charged groups, thus minimizing dipole–dipole interactions with foulants and enhancing antifouling performance.^40–42^ Type II photoinitiators such as benzophenone have been widely used for UV-induced graft-polymerization.^43^ However, benzophenone is associated with toxicity, coloring of final products, and poor water solubility, which limits its applicability for charged monomers.^44^ In this work, type I photoinitiators BAPO and LAP were employed. BAPO is less toxic than benzophenone but only sparingly water-soluble, whereas LAP is water-soluble and biocompatible.^45–47^ Both exhibit photobleaching behavior that preserves membrane color and allows deeper light penetration into the porous structure.^38^ The use of LAP therefore enables a greener grafting process. Moreover, grafting at 365 nm permits the use of energy-efficient LED light sources, further improving the environmental sustainability of the method. The resulting modified membranes were evaluated for their physicochemical properties, permeability and antifouling properties. In addition, the adsorption of contaminants was assessed using dyes as model contaminants.

## Experimental Section

### Chemicals and Materials

All standard chemicals were purchased as reagent grade and used without further purification prior to use. Solvents were used as HPLC grade unless otherwise stated. PAN membranes were prepared according to Scharnagl and Buschatz.^10^ Phenylbis­(2,4,6-trimethylbenzoyl)­phosphine oxide (BAPO), 2-bromoethanol, sodium 2-bromoethanesulfonate and 2-(dimethylamino)­ethyl methacrylate (DMAEMA) were purchased from abcr. Lithium phenyl­(2,4,6-trimethylbenzoyl)­phosphinate (LAP), 1,4-diazabicyclo[2.2.2]­octane (DABCO) and 2-methacryloyloxyethyl phosphorylcholine (MPC) were purchased from bldPharm. Sodium chloroacetate, vinylbenzyl chloride, dimethylamine solution and [2-(methacryloyloxy)­ethyl]­trimethylammonium chloride (METAC) solution were purchased from Sigma-Aldrich. Phosphate-buffered saline (PBS) with a final concentration of 140 mM NaCl, 10 mM phosphate, 2.7 mM KCl, and a pH adjusted to 7.4 was prepared as a stock solution. Microorganisms *S. aureus* (strain ATCC29213) was purchased from American Type Culture Collection.

### Membrane UV-Grafting

The desired monomer and the photoinitiator (LAP or BAPO) were dissolved in demineralized water. PAN membrane was added to the solution and was degassed with nitrogen for 20 min. The soaked PAN membrane was transferred to a new vial and was irradiated with UV light (365 nm, 30 W) for 1 h (methacrylate monomers) or 2 h (styrene monomers) at room temperature. The modified PAN membrane was subsequently cleaned with demineralized water (10 mL) in an ultrasonic bath three times 10 min and dried in vacuo at 50 °C. The specific reaction conditions for each modification can be found in the Supporting Information.

### Membrane Postmodification

PAN-*g*-VBD or PAN-*g*-DMAEMA was immersed in either H_2_O_2_ (30%), sodium chloroacetate solution (1 M), bromoethanol (neat) or 2-bromoethanesulfonate solution (1 M) for 24 h at 50 °C. The modified PAN membrane was subsequently cleaned with deionized water (10 mL) in an ultrasonic bath three times 10 min and dried *in vacuo* at 50 °C. The specific reaction conditions for each modification can be found in the Supporting Information.

### Infrared Spectroscopy

Infrared spectra were recorded with an attenuated total reflectance Fourier Transform infrared system (ATR-FTIR), model “IRAffinity-1S” from Shimadzu (Kyoto, Japan) using a “Quest” ATR accessory from Specac. The spectral range was set at 4000–500 cm^–1^ with a resolution of 0.5 cm^–1^ in absorbance mode. The spectra were processed with OriginPro 9 (2021) software.

### SEM/EDX Analysis

Scanning electron microscopy (SEM) was used to investigate the membrane morphology. SEM images of surfaces and cross-fractured specimens were recorded on a Merlin SEM (Zeiss, Jena, Germany) at accelerating voltages of 1.5–3 keV using an InLens secondary electron detector. Before measurement, the samples were dried under vacuum at 50 °C for 48–72 h and were sputter-coated with 1–1.5 nm platinum using a CCU-010 coating device (Safematic, Zizers, Switzerland). The pore size and porosity of the membrane surface were analyzed with the software IMS (Imagic Bildverarbeitung AG, Opfikon, Switzerland). The medium pore diameter (pore size) and the relative amount of pores on the surface (surface porosity) were determined. Four SEM images (from different areas) with a magnification of 100k (resolution of 1.117 nm) were used to evaluate each sample. Pores with an area smaller than 3 nm^2^ were excluded from the analysis. The values are given as the mean value ± standard deviation. Energy-dispersive X-ray spectroscopy (EDX) was used to investigate the membrane composition and the distribution of polymer in and on the membrane. EDX was measured with an accelerating voltage of 5 keV, a probe current of 150 pA and a working distance of 5.6–5.8 mm. Signals were detected by using an X-Max Extreme as a primary and an X-Max 150 as a secondary EDX detector. Before measurement, the samples were dried under vacuum at 50 °C for 48–72 h and were sputter-coated with 1.5 nm platinum using a CCU-010 coating device (Safematic, Zizers, Switzerland).

### Contact Angle Measurements

Contact angles were acquired with an OCA 20 goniometer from DataPhysics (Filderstadt, Germany) equipped with two automated dispensing units for different liquid probes, a high-speed video system with CCD-camera, measuring stage and halogen-lighting for static and dynamic contact angle measurements. For evaluation, independent triplicate measurements at three different points of the surface were done. Contact angles were measured with deionized water using the static sessile drop method with a dispensing volume of 2 μL. The dispensing rate of the automatic syringe was set at 1 μL min^–1^. To obtain the contact angle a short video of 10 s with a frequency of 10 Hz was recorded. The contact angle was then determined as the mean value of the first three contact angles. The obtained angle was calculated with the OCA software.

### AFM Measurements

The surface roughness of the samples was determined with the Multimode 8 atomic force microscope from Bruker in the PeakForce QNM mode. ScanAsyst Air with a spring constant of 0.4 N/m and a radius of 2 nm was used as a tip. The maximum force was 500 pN with a frequence of 1 kHz and a scanning velocity of 0.47 Hz. The arithmetic average roughness (*R*_a_) was then determined as the mean value of the surface scans in an area of 9 μm^2^.

### Molecular Weight Cut-Off

Retention of four different PEG solutions (i.e., Molar mass of PEG 44, 82.8, 141.7, 219.4, 250, 351 kDa) was analyzed. For the PEG 44, 82.8 and 141.7 kDa, 50 mg each were combined and dissolved in 500 mL Milli-Q water. For the other PEG′s i.e., 219.4, 250 and 351 kDa, 50 mg each were dissolved in 500 mL Milli-Q water. Membrane pieces with a diameter of 2.0 cm were used (active membrane surface 1.68 cm^2^). Before the adsorption tests, water was filtered through the membranes at 2 bar transmembrane pressure for 1.5 h to avoid swelling during the measurements. Afterward, the PEG solutions were filtered through the membranes using an Amicon cell in dead-end mode (while stirring) at 2 bar transmembrane pressure. Samples of the permeate and the retentate were taken after 1.5 h. Additionally, a sample of the feed solution was taken. The concentration of the PEG in the respective sample was determined using GPC (PSS Polymer Standards Service). Two samples of each modified membrane were measured. The values are given as the mean value. With eq 1 it is possible to calculate the molecular weight cutoff, where Ret is the Retention, *c*(P) the Permeate concentration, *c*(F) the Feed concentration and *c*(R) the Retentate concentration.

1 $$\mathrm{Ret}=1-\frac{\left(c\left(P\right)+c\left(F\right)\right)}{2\cdot c\left(R\right)}\cdot 100$$

### Pure Water Permeability

The permeance was measured using an inbuilt (Hereon) permeance measurement device in dead-end mode. It was measured on a circular piece of the membrane with a diameter of 2.0 cm corresponding to an active area (*A*) of 1.68 cm^2^. Ultrapure water was used to measure the permeance. The measurement was done multiple times to see if the results were reproducible. The permeance (*P*) was calculated using the following eq 2, where Δ*V* is the Volume difference, Δ*p* the pressure difference and Δ*t* the time difference.

2 $$P=\frac{\Delta V}{\Delta p\cdot \Delta t\cdot A}$$

### Zeta Potential Measurement

The ζ potential is an indication of the surface charge of the membrane. It was measured using the SurPASS Eco 3 from Anton Paar (Graz, Austria). The streaming potential method was used to measure the ζ potential. The electrolyte solution used was 0.01 M NaCl solution and the pH was adjusted using 0.05 M NaOH and 0.05 M HCl. All solutions were prepared using ultrapure Milli-Q water. Before starting the ζ potential measurement, the membranes were rinsed multiple times with the electrolyte solution. The measurements were performed in the pH range of 9 to 3. At each pH, the ζ potential was measured four times.

### UV/vis Spectroscopy

UV–vis spectra were obtained on a Genesys 10S spectrophotometer from Thermo Scientific (Waltham) using Visionlite software for analysis.

### Static Dye Adsorption

The adsorption of two dyes (i.e., a negatively and a positively charged dye) was analyzed. orange II was chosen as the negatively charged dye and methylene blue was chosen as the positively charged dye. A 50 μM solution of orange II and a 25 μM solution of methylene blue were prepared separately. Thus, the orange II solution contained 17.6 mg/L orange II and the methylene blue solution contained 9.8 mg/L methylene blue. Membrane pieces with a diameter of 2.0 cm were used (active membrane surface 3.14 cm^2^ for the static test). For the static test membranes from the water flux measurement were placed in a vial with 10 mL of the described dye solutions. The vials were left to stand for 7 days and afterward, the amount of unabsorbed dye was determined via UV–vis. The amount of absorbed dye can be calculated using eq 3, where *N* are the molecule, *A* the area, *C*_0_ the initial dye concentration, *C* the measured dye concentration, *N*_A_ the Avogadro constant and *V* the Volume.

3 $$\frac{N}{A}=\frac{\left(C_{0}-C\right)\cdot N_{A}\cdot V}{A}$$

### Dynamic Dye Adsorption

For the dynamic dye adsorption membrane pieces with a diameter of 2.0 cm were used (active membrane surface 1.68 cm^2^). Before the dynamic adsorption of orange II (250 mL, 2.0/0.2 μM) and methylene blue (250 mL, 1.0 μM) water was filtered through the membrane for 1 h at 1 bar transmembrane pressure to avoid swelling. Then the dye solutions with the given concentration and amount were filtered through the membrane in an Amicon cell in dead-end mode (while stirring) at 1 bar transmembrane pressure. Ten mL samples of the permeate were taken until the feed concentration was reached. Additionally, in the beginning, one sample of the feed solution and in the end one sample of the retentate were taken. The concentration of the dye in the respective sample was determined using UV/vis spectroscopy (GENESYS 10S spectrophotometer, Thermo Scientific, Waltham, MA). The absorbance of methylene blue was measured at 665 nm and that of orange II was measured at 482 nm. The absorbance of each sample was compared with a previously measured calibration curve to calculate the concentration. Two samples of each modified membrane were measured in the dynamic experiment and one each in the static experiment. The values for the dynamic experiment are given as the logistic fit of the mean value.

### Determination of Antimicrobial Activity (ASTM E2149-13a)^48^

The antimicrobial evaluation was performed using a modified ASTM E2149-20 assay.^48^ All membranes were treated with 70 vol % isopropyl alcohol and dried at room temperature under laminar airflow prior to testing. The test microorganism *S. aureus* (ATCC 29213) was cultured on Columbia agar for 12 h. The cultures were harvested, suspended, and diluted with sterile NaCl solution (0.9 wt %) to a concentration of 10^5^ CFU/mL. Membrane samples (1.0 cm^2^) were incubated with 2 mL of the bacterial suspension (10^5^ CFU/mL) in sterile tubes and shaken at 300 rpm for 2 h at 37 °C. After incubation, the solutions and its serial dilutions were plated on Columbia agar (100 μL of 10^5^, 10^4^, 10^3^, and 10^2^ CFU/mL) and incubated for 17 h at 37 °C. After incubation, colonies were counted and reported as mean values.

## Results and Discussion

### Membrane Fabrication

The UV-induced graft-polymerization developed in this work is a gentle method in which the bulk material remains largely intact. Irradiation of a photoinitiator produces radicals that abstract hydrogen atoms from the membrane surface and thus serve as a starting point for the attachment of monomers. The modification process is shown schematically in Figure 1.

**Figure 1:**
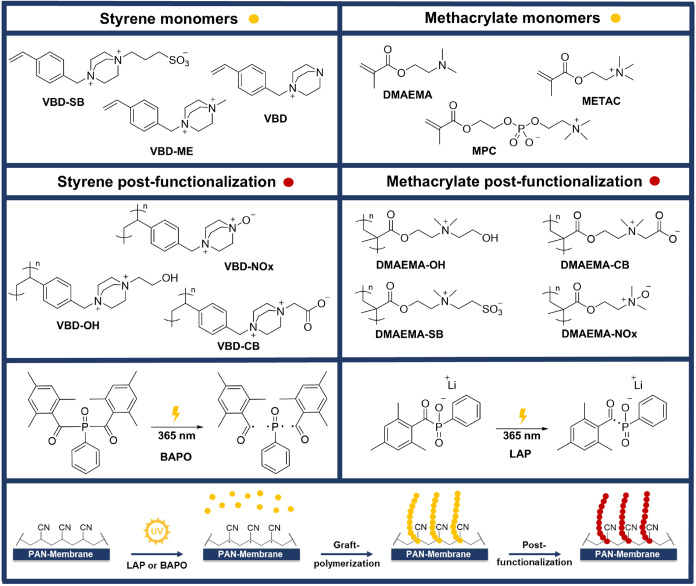
UV-induced grafting of charged and zwitterionic polymers from PAN membranes using LAP or BAPO as photoinitiators. Grafted brush polymers were prepared either by direct graft- polymerization of charged or zwitterionic monomers (yellow) or by postfunctionalization of grafted brush polymers (red) based on DMAEMA or VBD.

Irradiation was performed using an energy efficient LED light source with a narrow emission spectrum at 365 nm. UV-induced graft-polymerization on PAN ultrafiltration membranes have been performed with benzophenone as a type II photoinitiator.^43^ In this work, acylphosphine oxides such as BAPO (IRGACURE 819) and LAP are used as type I photoinitiators. Radicals are formed by α-cleavage from the excited triplet state, which is achieved after photoexcitation and intersystem crossing.^49^ A major advantage of these initiators is their absorption in the near UV (350–400 nm), high quantum yield of radicals, high reactivity of the resulting benzoyl and phosphinoyl radicals, fast photolysis, and good solubility. The latter property is particularly important for the polymerization of highly polar charged monomers. In addition, acylphosphine oxides exhibit photobleaching, in which the chromophores are destroyed during irradiation, resulting in a colorless reaction mixture. This allows deeper light penetration into the coating and promotes complete curing.^38^ A disadvantage is the high cytotoxicity of certain acylphosphine oxides such as BAPO.^50^ Interestingly, the corresponding acylphosphine oxide salts have been found to be nontoxic. LAP, for example, has been shown to have good biocompatibility.^47^

DMAEMA (2-(dimethylamino)­ethyl methacrylate) and VBD (1-(4-vinylbenzyl)-1,4-diazabicyclo[2.2.2]­octan-1-ium chloride) were selected as starting materials. Many functional groups can be introduced via the tertiary amine present in both compounds by nucleophilic substitution or oxidation. Furthermore, the resulting polymers are quite stable.^51^

The functional groups attached to the polymer brushes were selected along the following lines. Antimicrobial contact-active polymer brushes were prepared by the introduction of quaternary ammonium groups.^52^ The antibacterial properties of these brushes depend on the density of positively charged groups.^53–55^ In addition, the positively charged polymer brushes may not only eliminate microorganisms, but might also adsorb negatively or block positively charged contaminants in wastewater, such as dyes or drugs. For the synthesis of these polycationic brushes, we used two different protocols (methods A and B in Figure 2). Method A: The ammonium derivatives METAC and VBD-ME were used to grow the polycationic brush polymers from the membrane surface. Method B: The tertiary amines DMAEMA and VBD were used for grafting polymers with tertiary amine groups as precursor for postfunctionalization by nucleophilic substitution with 2-bromoethanol to form quaternary ammonium groups containing additional hydroxyl groups. The idea was to combine the contact-active effect of the ammonium group with an antiadhesive component of the well-hydrated hydroxyl group.

**Figure 2:**
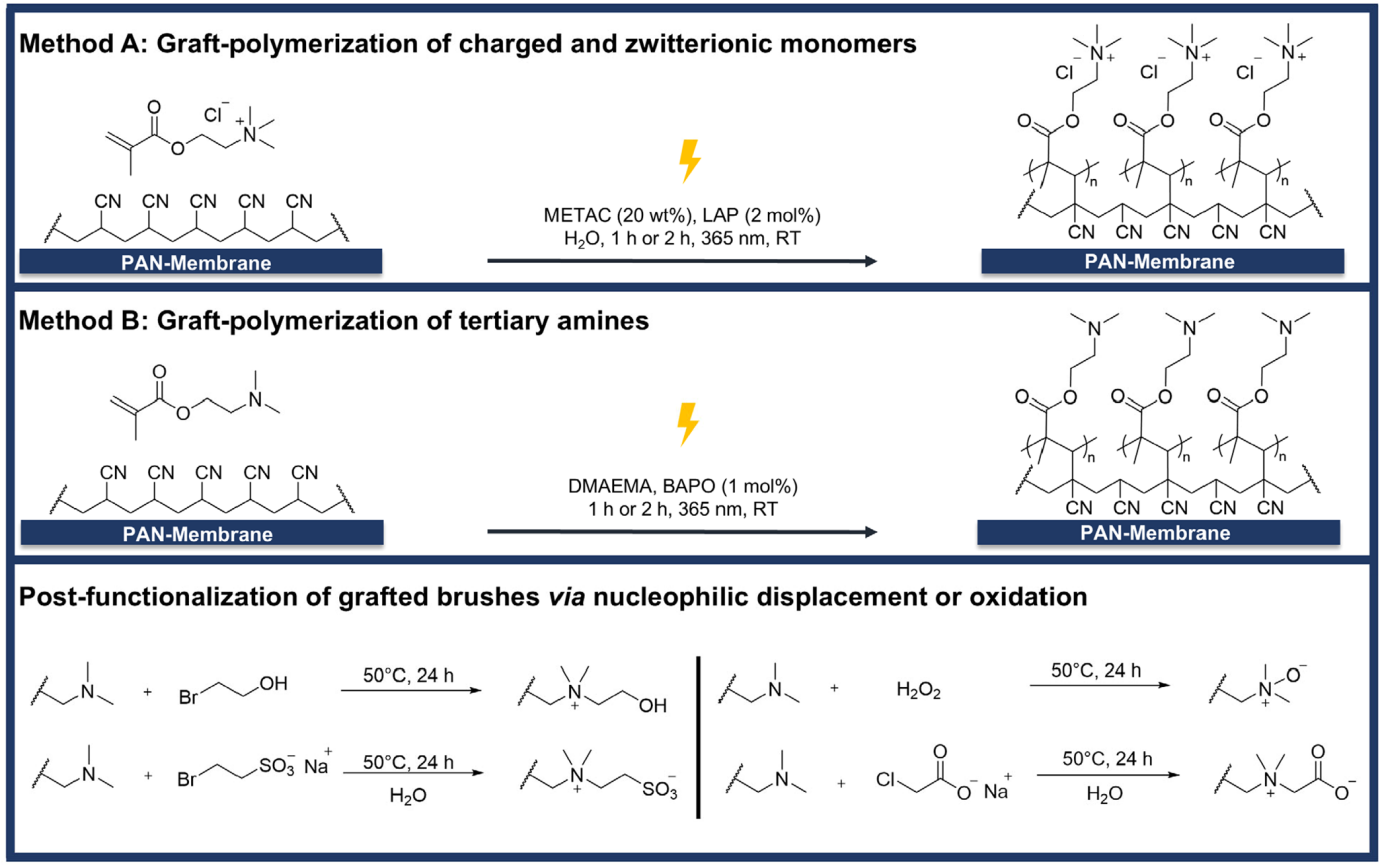
Reaction conditions for UV-induced graft-polymerization: Method A, optimized for nonpolar monomers (e.g., DMAEMA), and Method B, applied to hydrophilic monomers (e.g., METAC). Reaction schemes for the postmodification of polymer brushes with tertiary amines to obtain ammonium alcohols, sulfobetaines, carboxybetaines, and *N*-oxides.

Zwitterionic polymer brushes based on carboxybetaines, sulfobetaines, phosphobetaines and *N*-oxides can prevent the accumulation of biomolecules and microorganisms on surfaces and can also bind certain wastewater contaminants.^41,56,57^ Most zwitterionic polymer brushes containing *N*-oxides (NOx), carboxybetaines (CB), and sulfobetaines (SB) were prepared by Method B in Figure 2 and thus by grafting of DMAEMA and VBD and subsequent postgrafting functionalization. Only sulfobetain VBD-SB and phosphobetain MPC were grafted directly from the PAN membranes following Method A in Figure 2.

To reduce harmful solvents, BAPO was found to dissolve well in nonpolar monomers (e.g., DMAEMA), eliminating the need for additional solvents. Therefore, Method A is applied for liquid, nonpolar monomers. For solid, water-soluble monomers (e.g., charged or zwitterionic monomers), Method B was developed using water as a solvent and the water-soluble initiator LAP.

UV-induced graft-polymerization was performed in the absence of oxygen.^58^ Briefly, the PAN membrane was cut into 1 cm^2^ pieces and mixed with a solution of photoinitiator and monomer. This solution was degassed and the mixture was then irradiated at a wavelength of 365 nm in the photoreactor. The UV-induced graft-polymerization duration was adjusted according to monomer reactivity. Methacrylate monomers, being more reactive, were polymerized for 1 h, whereas the less reactive styrene derivatives required 2 h of UV exposure to achieve sufficient grafting.

Both methods A and B have advantages and drawbacks: Direct graft-polymerization of charged or zwitterionic monomers (Method A) is a simple and effective method that allows monomers to be grafted directly onto the membrane surface in one step. However, the chemical compatibility of certain monomers with the reaction conditions, such as pH, solvent, or light sensitivity, can limit this approach. In addition, some zwitterionic or functional monomers require time-consuming syntheses, which makes direct grafting impractical. An advantage is that the density of functional groups is homogeneous among different graft polymers. However, the polymer length might vary significantly. In contrast, postmodification (Method B) allows the use of commercial monomers for graft polymerization. Then, a second reaction step is performed to introduce the desired functionality. Although this increases the number of steps, it offers greater versatility, particularly for sensitive or synthetically challenging structures. Postfunctionalization is thus an attractive method for generating a library of functional polymers. The advantage is that all final brush layers are prepared from the same precursor and have thus comparable loading density. However, the density of functional groups might be different due to incomplete postgrafting functionalization. For the comparison of membrane properties it is important to keep these limitations in mind.

The modification process was optimized with respect to monomer concentration and reaction time in each case to achieve densely functionalized surfaces while maintaining adequate permeance of around 100 L m^–2^ h^–1^ bar^–1^ to ensure sufficient water flow through the membrane.

### Membrane Characterization

The physicochemical properties of the modified membranes were analyzed by measurement of the pH-dependent surface ζ potential, ATR-FTIR and SEM-EDX. An example is depicted in Figure 3 for the analysis of a polysulfobetaine-modified PAN membrane (PAN-*g*-VBD-SB). The characterization of the other materials is available in the Supporting Information.

**Figure 3:**
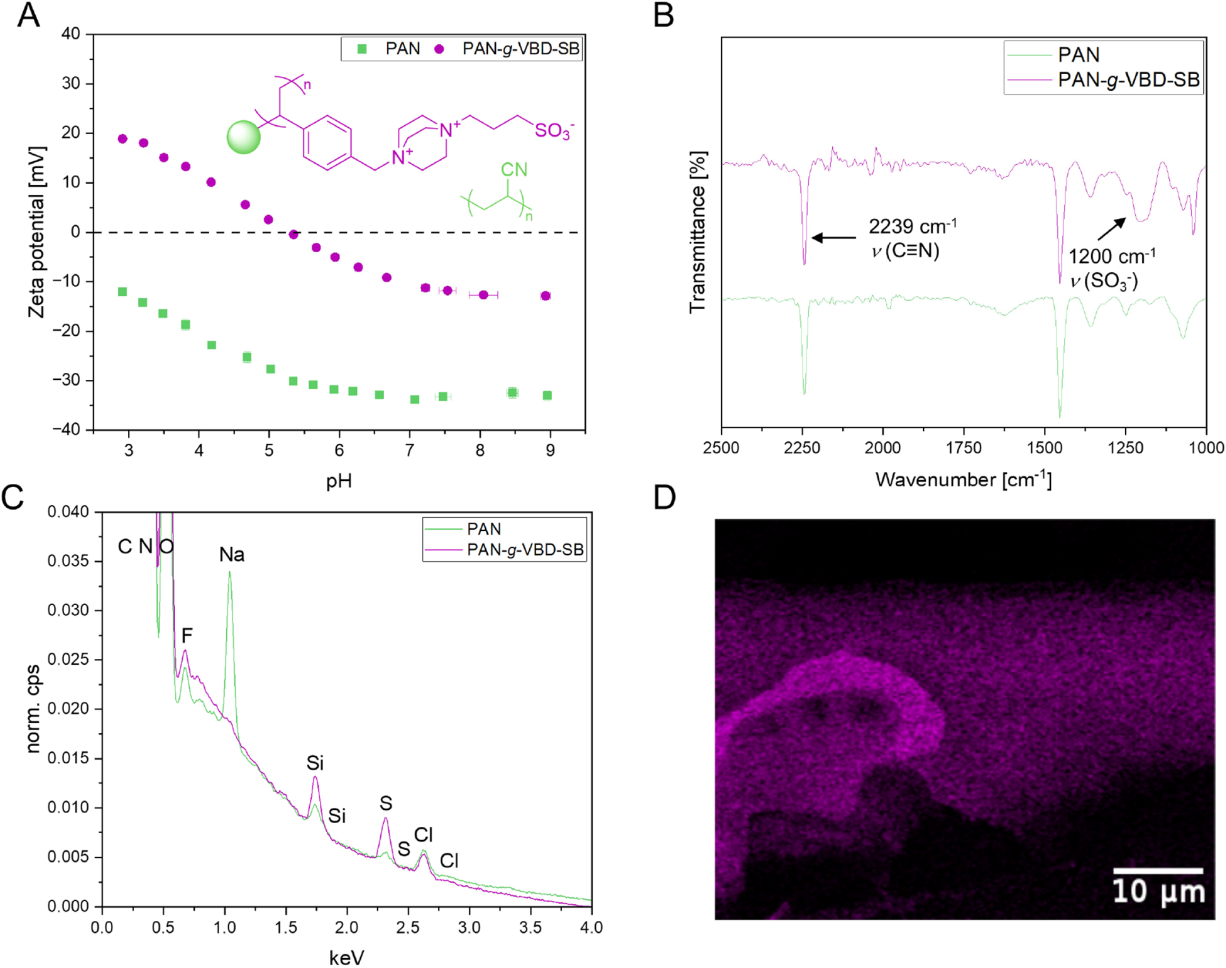
Surface ζ-potential, ATR-FTIR and EDX-spectra of the PAN-*g*-VBD-SB modified PAN membrane. (A) Surface ζ-potential of PAN-*g*-VBD-SB compared to pristine PAN membrane at different pH values. (B) ATR-FTIR spectra of PAN-*g*-VBD-SB compared to pristine PAN. Selected bands of the polymer brushes are highlighted. (C) EDX survey spectra of PAN-*g*-VBD-SB compared to pristine PAN membrane surface. (D) Cross-fracture of PAN-*g*-VBD-SB with sulfur EDX mapping in purple.

The measurement of the surface ζ potential (Figure 3A) reveals higher values in a pH range from 3 to 9 for PAN-*g*-VBD-SB compared to pristine PAN. This reflects the overall positive charge of the brush polymer PAN-*g*-VBD-SB and agrees well with the data obtained for similar mixed zwitterionic/cationic brush polymers on other base materials.^23^

Fourier-transform infrared spectroscopy (FTIR, Figure 3B) confirmed the presence of sulfonate groups and thus the successful grafting of polysulfobetaine (PAN-*g*-VBD-SB) onto the membrane surface. The characteristic nitrile band of polyacrylonitrile appears at 2242 cm^–1^, indicating the intact base material.

Scanning electron microscopy coupled with energy-dispersive X-ray spectroscopy (SEM-EDX, Figure 3C,D) provided insights into surface and cross-fracture morphology as well as elemental composition. The elemental mapping of sulfur via EDX confirmed the presence of sulfobetaine throughout the membrane structure, indicating that the grafting process was not limited to the surface but extended into the internal pore network. The EDX spectra and the corresponding line scans for PAN-*g*-DMAEMA-SB and PAN-*g*-VBD-SB are provided in the Supporting Information (S10–S13).

The images of surface and cross-fractured specimens (Figures S5–S7) confirm that the pores remain visible after the grafting process and are not blocked by the grafted polymers. The values for pore size and porosity for each modification are available in the Supporting Information (Table S1). The measured pore size and porosity of the pristine PAN membrane were determined to be 8.4 nm and 1.79%, respectively. For modifications based on a vinylbenzene scaffold, the pore size ranged from 8.1 to 8.5 nm, while the surface porosity varied between 0.88 and 1.85%. In contrast, membranes functionalized with methacrylate monomers exhibited a broader pore size distribution, ranging from 8.9 to 11.1 nm, with surface porosity values between 1.65% and 5.40%. This observation can be explained by several factors. The grafting occurs primarily on the membrane surface and pore walls without completely sealing the pores, preserving the overall pore structure. In addition, during the grafting process, solvent interactions and swelling might induce temporary structural changes, which, upon drying, result in a stabilized but slightly expanded pore network. Furthermore, the introduction of functional groups such as sulfonates, phosphate diesters, or carboxybetaines may lead to electrostatic interactions that influence polymer chain conformation, potentially increasing the effective pore size. Overall, the results indicate that the applied grafting method does not obstruct the membrane pores but instead leads to a moderate increase in pore size and porosity.

A comparison of the surface ζ potential reveals significant differences in surface charge between the unmodified PAN membrane and the modified membranes. The results are presented in Figure 4, comparing pristine PAN with vinylbenzen-based (Figure 4A) and methacrylate-based (Figure 4B) modifications.

**Figure 4:**
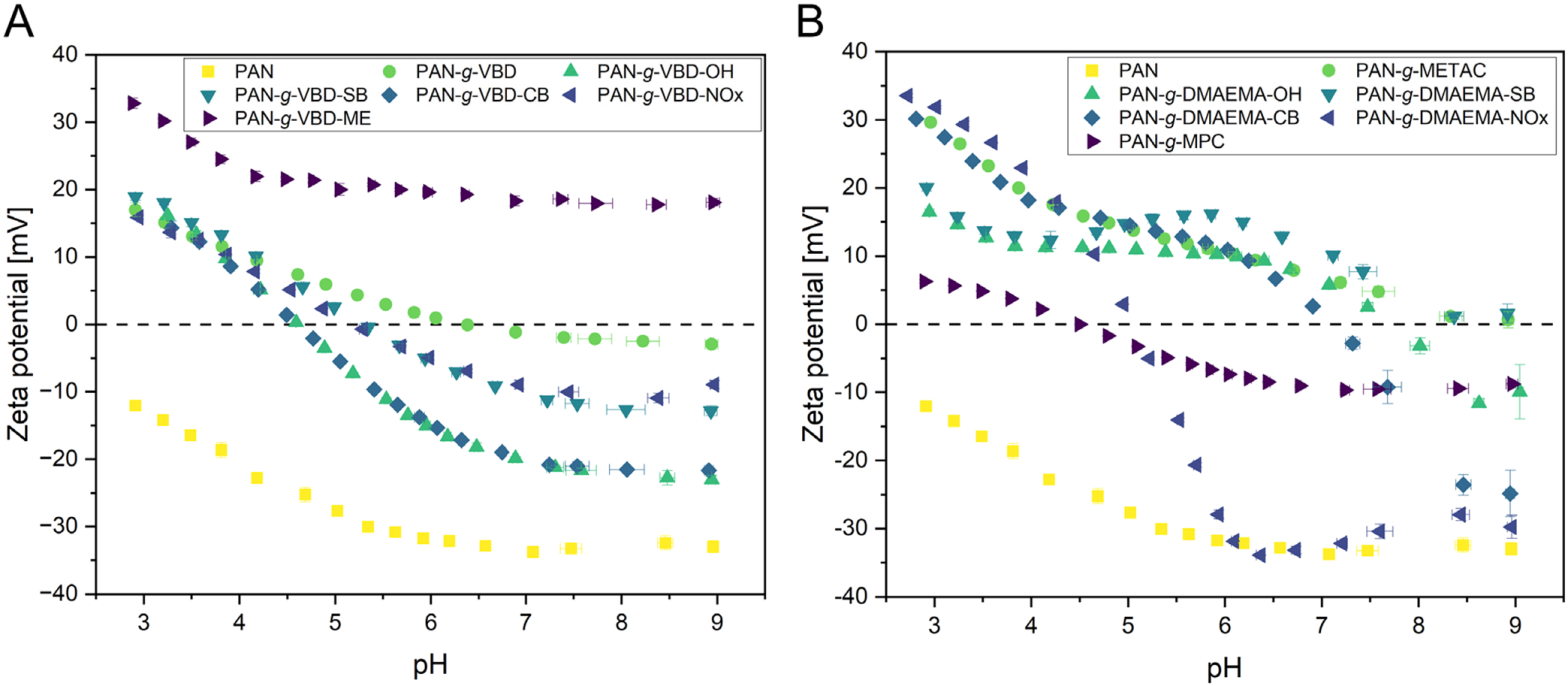
Surface ζ potential of pristine PAN and modified membranes at different pH values. (A) Modifications based on a vinylbenzene scaffold; (B) modifications based on a methacrylate scaffold.

The pristine PAN membrane is negatively charged across the entire pH range due to hydroxide adsorption.^59^ All modified membranes have more positive ζ potentials than pristine PAN. PAN-*g*-VBD-ME and PAN-*g*-METAC are positively charged across the entire pH range measured. PAN-*g*-VBD-ME is slightly more positive than PAN-*g*-METAC because it has two positively charged nitrogen atoms. Many vinylbenzene or methacrylate modifications, have pH-dependent transitions from positive to negative surface ζ potentials. These transitions reflect the protonation or deprotonation of tertiary amines, alcohols, sulfobetaines, carboxybetaines, and *N*-oxides. Most of the surface ζ potential values reflect thus the chemical modifications of the membranes quite well. PAN-*g*-DMAEMA-SB is an exception: as a zwitterion, a pH dependent transition of the surface ζ potential to negative values was expected. However, the surface ζ potential of this modification was positive over the measured pH range suggesting a polycationic brush layer. This unexpected profile is a consequence of noncomplete postgrafting functionalization of PAN-*g*-DMAEMA with 2-bromosulfonate. The latter tend to elimination and NMR analysis confirmed the incomplete conversion in a model reaction in solution under identical postfunctionalization conditions (Figure S23). The preparation of the zwitterionic PAN-*g*-DMAEMA-SB was thus not successful and the positive surface ζ potential values over a broad pH range reflect the incomplete postfunctionalization leaving a large number of tertiary amine groups on the surface.

Overall, the pH dependent surface ζ potentials reflected the expected chemical properties of the brush polymers and thus the successful grafting in most cases. However, the magnitude of the charge effects cannot be compared quantitatively among different materials due to possible differences in grafting efficiency or postgrafting functionalization.

Atomic force microscopy (AFM) was utilized to examine the topography and arithmetic average roughness of the membranes on the nanoscale (Table S1). Pristine PAN exhibits a surface roughness of 3.38 nm. The vinylbenzen derivatives display roughness values ranging from 2.44 to 4.01 nm, while the methacrylate derivatives have roughness values between 2.70 and 3.71 nm. Notably, the *N*-oxide modifications of both vinylbenzen (4.01 nm) and methacrylate (3.71 nm) derivatives have the highest roughness values. This might be attributed to the postgrafting treatment with hydrogen peroxide, a strong oxidizing agent that might alter the surface properties. As a result, irregularities form, leading to increased roughness (Figures S8–S9). It is notable in this context, that membranes with lower surface roughness and higher water wettability are generally considered less susceptible to fouling.^60–62^

To investigate changes in surface hydrophilicity, contact angle measurements were performed. Low contact angles indicate increased hydrophilicity, which enhances the formation of a hydration layer on the membrane surface that acts as a physical barrier, reducing protein and bacterial adhesion.^63^ The water contact angle (WCA) measurements provided indirect evidence of successful surface modifications, as all modified membranes revealed changes compared to the pristine PAN membrane. The dynamic WCA was measured over 1.5 s, and the mean values are presented in the Supporting Information (Table S1). The pristine PAN membrane exhibited a WCA of 44.8°. Among the modified membranes, PAN functionalized with VBD-NOx had the lowest WCA of 34.3 °. PAN functionalized with DMAEMA–OH had the highest WCA of 61.0°. Accordingly, the *N*-oxide functionalized membranes are expected to show the best antifouling performance among the tested materials, due to their highest hydrophilicity. The pure water permeance of the membranes was evaluated in dead-end mode to assess their suitability for filtration and the results are depicted for vinylbenzen-based (Figure 5A) and methacrylate-based (Figure 5B) modifications.

**Figure 5:**
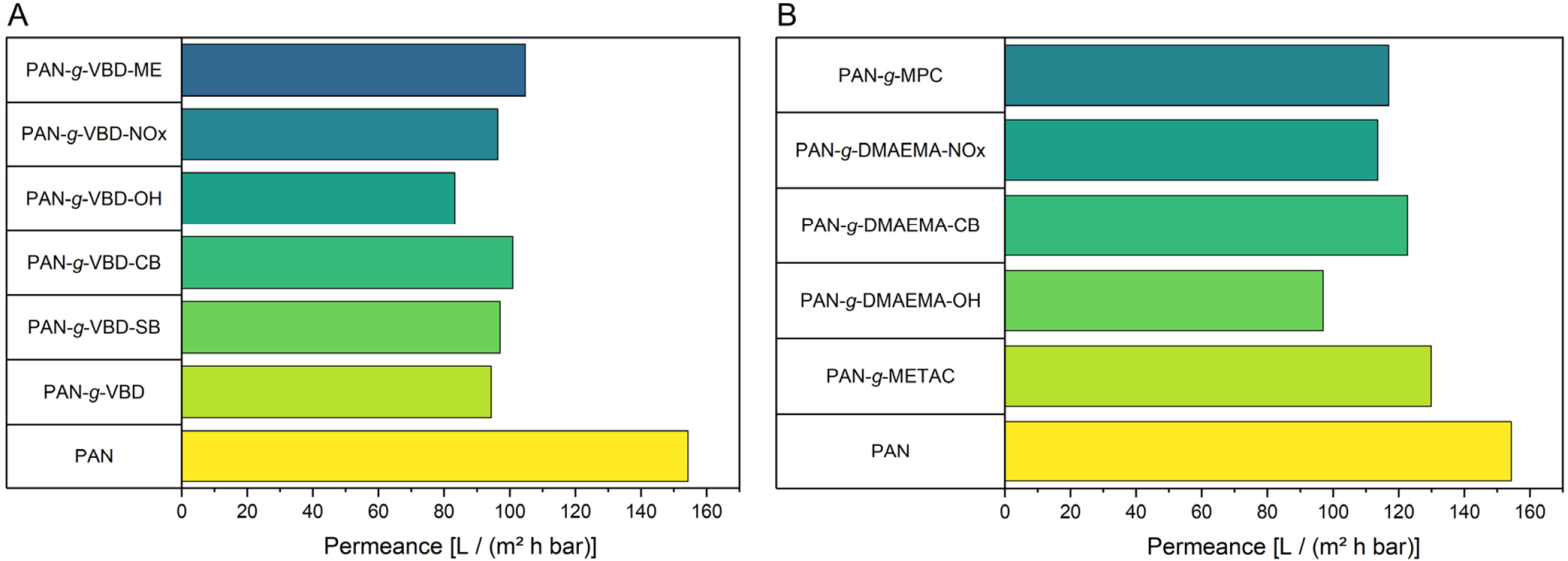
Pure water permeance of pristine PAN and modified membranes at different pH values. (A) Modifications based on a vinylbenzene scaffold; (B) modifications based on a methacrylate scaffold.

The pristine PAN membrane revealed the highest permeance with 154 L/(m^2^ h bar), while all modified membranes had a slightly reduced water permeance due to the incorporation of different functional groups. Swelling of the membrane reduces its permeance because water uptake causes the polymer chains to expand, partially blocking pores and increasing the tortuosity of the transport pathways. This effect is more pronounced for cationic and zwitterionic membranes, as their charged groups strongly bind water, further enhancing swelling and slightly decreasing water flux compared to less hydrophilic modifications.

The molecular weight cutoff (MWCO) of the membranes remained largely unchanged after modification (Table S1). The pristine PAN membrane had a MWCO of 351 kDa, while the modified membranes showed MWCO values ranging from 351 to 279 kDa. While the SEM images in the dry state suggested a structure with enlarged pores, the MWCO analysis in the hydrated state indicates a slightly smaller pore size of the modified materials compared to the PAN membrane, which can be attributed to polymer swelling.

### Static and Dynamic Dye Adsorption

Orange II and methylene blue were used as model substances to evaluate the dye adsorption of the modified membranes. Orange II is an anionic azo dye containing a sulfonate group, while methylene blue is a cationic phenothiazine dye. These structural differences allow the investigation of selective retention of positively and negatively charged wastewater contaminants by the membrane. To assess the adsorption, a static dye adsorption test was first performed (Figure 6A,C), followed by measurements in the dead-end filtration mode (Figure 6B,D). Static dye adsorption serves as an indirect method to determine surface charge, complementing the surface ζ potential analysis. By examining the extent of dye uptake, valuable insights can be gained into the electrostatic interactions between the modified membrane surfaces and the charged dye molecules. To evaluate the membrane performance under more realistic conditions, dynamic dye absorption experiments were also performed.

**Figure 6:**
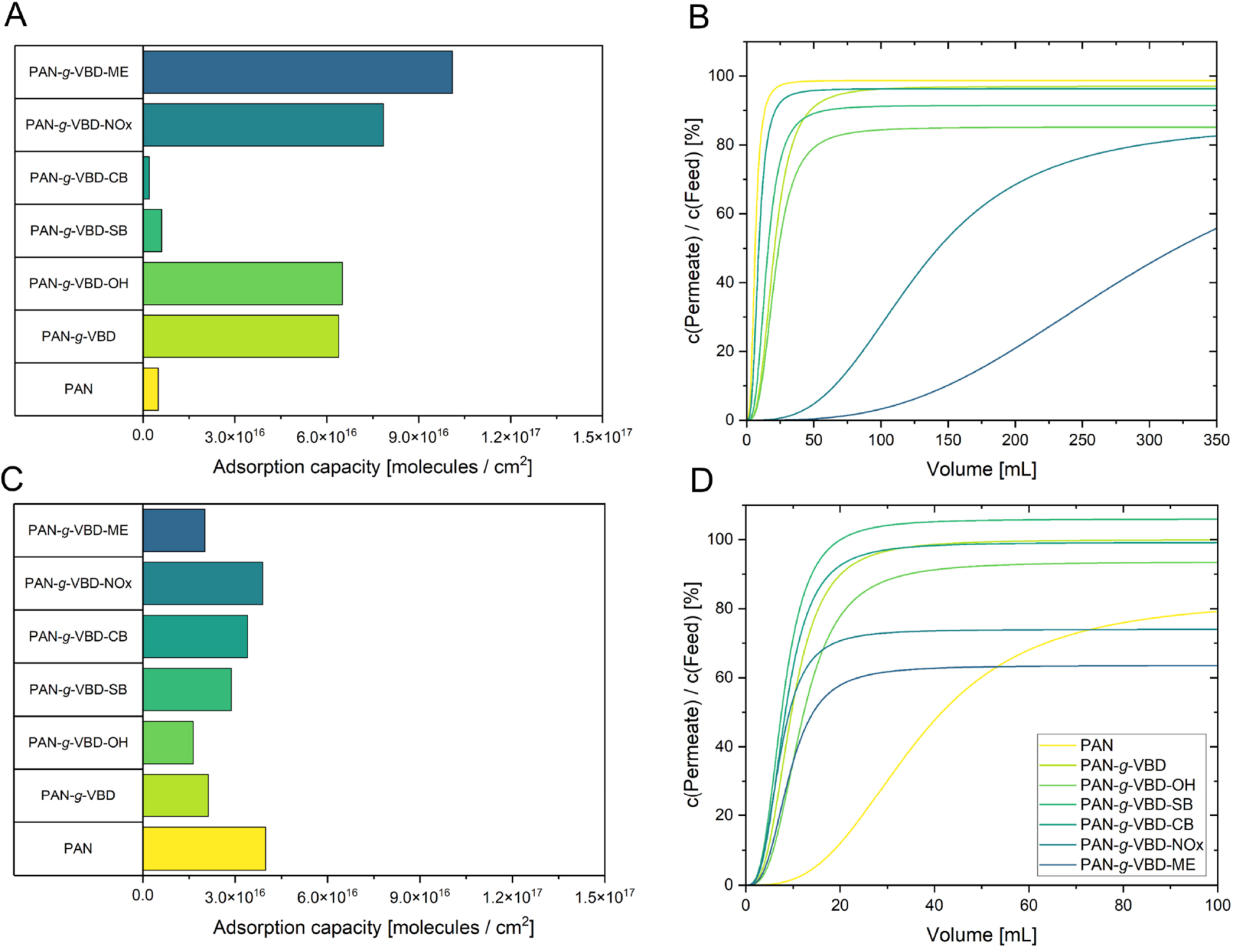
(A) static dynamic adsorption of orange II; (B) dynamic adsorption of orange II; (C) static adsorption of methylene blue; (D) dynamic adsorption of methylene blue on pristine PAN and the vinylbenzen-based modifications.

The adsorption of orange II and methylene blue to the membrane is strongly governed by electrostatic interactions and is in line with the surface ζ potential of the membranes. Pristine PAN shows negligible uptake of orange II due to electrostatic repulsion between the negatively charged membrane surface and the anionic dye. In contrast, positively charged vinylbenzen-based modifications significantly enhance orange II adsorption, with PAN-*g*-VBD-ME showing the highest adsorption capacity, consistent with its double cationic functionality and the strongly positive surface ζ potential. PAN-*g*-VBD-NOx, PAN-*g*-VBD, and PAN-*g*-VBD–OH also adsorb orange II but to a lesser extent. PAN-*g*-VBD-SB and PAN-*g*-VBD-CB do not adsorb orange II and are thus comparable to pristine PAN.

For methylene blue, the adsorption trend is reversed. Pristine PAN adsorbs high amounts due to its negative surface charge, whereas the overall positive charge of the vinylbenzene-based membranes led to relatively low amounts of dye adsorption. Interestingly, PAN-*g*-VBD-NOx led to comparable uptake of both dyes, indicating that nonelectrostatic interactions such as π–π stacking or hydrogen bonding may contribute in this case. The other VBD modifications adsorb methylene blue only to a limited extent.

Dynamic adsorption experiments confirmed the trends observed under static conditions. Pristine PAN did not retain orange II, whereas PAN-*g*-VBD-NOx and PAN-*g*-VBD-ME had the highest adsorption capacity prior to dye permeation through the membrane, consistent with the static results. For methylene blue, pristine PAN again showed the strongest uptake, while PAN-*g*-VBD-ME and PAN-*g*-VBD led to weaker retention, which may be attributed to a strong Donnan effect.^64^

For the methacrylate modifications, PAN-*g*-DMAEMA–OH and PAN-*g*-DMAEMA-CB showed the highest orange II adsorption, whereas PAN-*g*-METAC, PAN-*g*-DMAEMA-NOx and PAN-*g*-MPC adsorbed less of the anionic dye. The measurements were again performed under static (Figure 7A,C) and dynamic conditions (Figure 7B,D). Interestingly, despite their overall positive charge, the vinylbenzene-based modifications did not outperform the methacrylate-based ones in orange II adsorption. This may be due to differences in grafting density, steric hindrance, or polymer–matrix interactions, which could limit the accessibility of functional groups.

**Figure 7:**
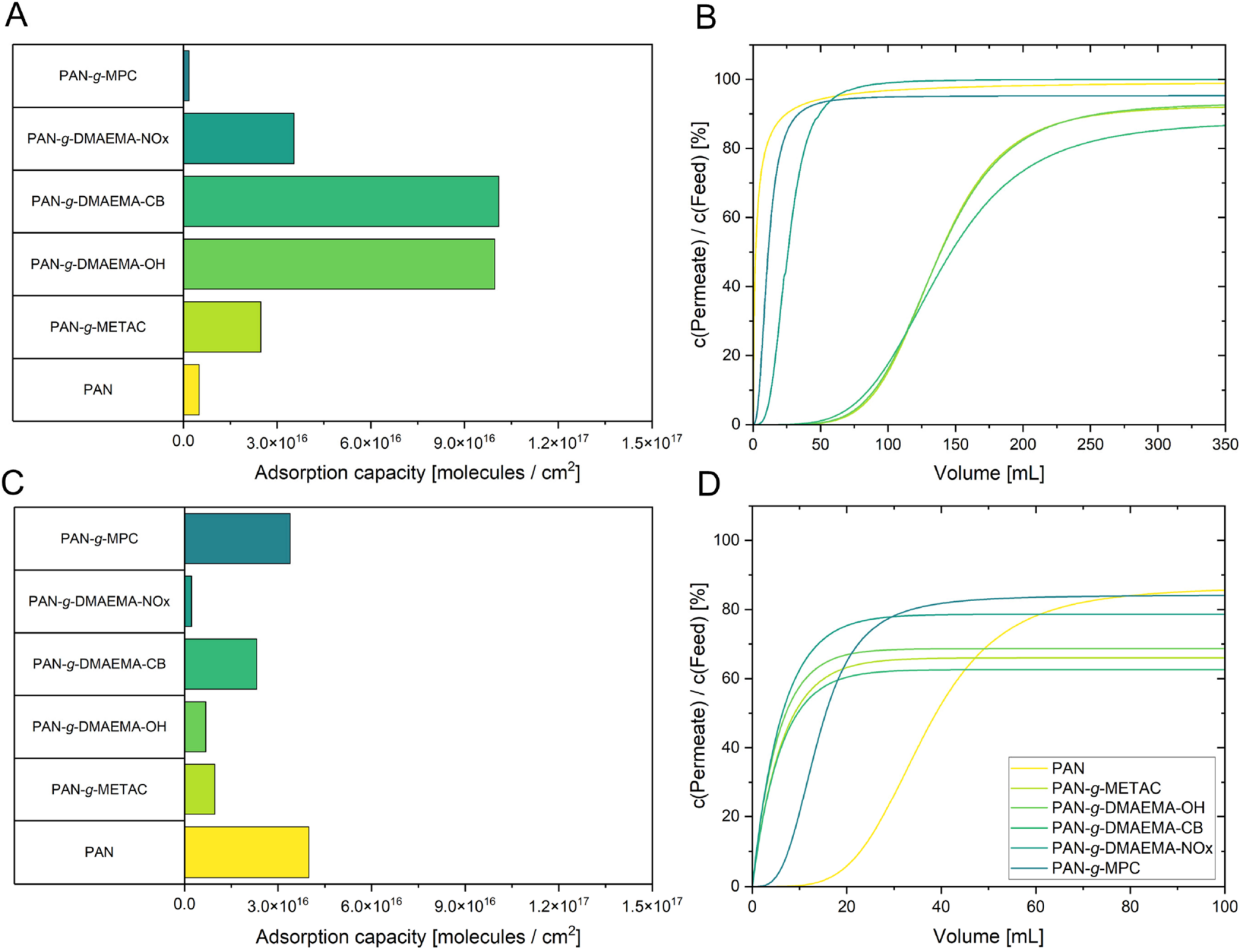
(A) Static adsorption of orange II; (B) dynamic adsorption of orange II; (C) static adsorption of methylene blue; (D) dynamic adsorption of methylene blue on pristine PAN and the methacrylate-based modifications.

For methylene blue, PAN-*g*-MPC led to the highest adsorption, consistent with its negative ζ potential, while all other methacrylate modifications showed little to no adsorption. In general, methacrylate modifications adsorbed less methylene blue than the vinylbenzen-based polymers, indicating that surface ζ potential alone does not fully explain the observed retention of charged dyes. In addition, specific structural features and surface accessibility likely play a role.

Dynamic adsorption experiments confirmed the static trends. PAN-*g*-METAC, PAN-*g*-MPC, PAN-*g*-DMAEMA-NOx, PAN-*g*-DMAEMA–OH, and PAN-*g*-DMAEMA-CB led to measurable orange II adsorption, whereas PAN-*g*-DMAEMA-NOx and PAN-*g*-MPC had almost no retention of the dye. For methylene blue, none of the methacrylate modifications showed significant uptake, though all demonstrated some degree of dye retention. Overall, both static and dynamic results suggest that PAN-*g*-VBD-ME, PAN-*g*-VBD-NOx, PAN-*g*-DMAEMA-CB, and PAN-*g*-DMAEMA–OH are the most promising candidates for the removal of negatively charged dyes, while pristine PAN remains best suited for positively charged dyes.

### Antibacterial Evaluation

The contact-biocidal efficacy of the various modified membranes was evaluated using a modified ASTM E2149-20 assay, a widely used method for assessing antibacterial materials. In this assay, pristine PAN and modified PAN membranes were incubated for 2 h with a bacterial suspension (10^5^ CFU/mL) at 37 °C. After incubation, aliquots of the suspension were diluted to 10^4^, 10^3^, and 10^2^ CFU/mL and plated onto Columbia agar. Following a further 17 h of incubation, the bacterial colonies were counted, and the results are shown in Figure 8.

**Figure 8:**
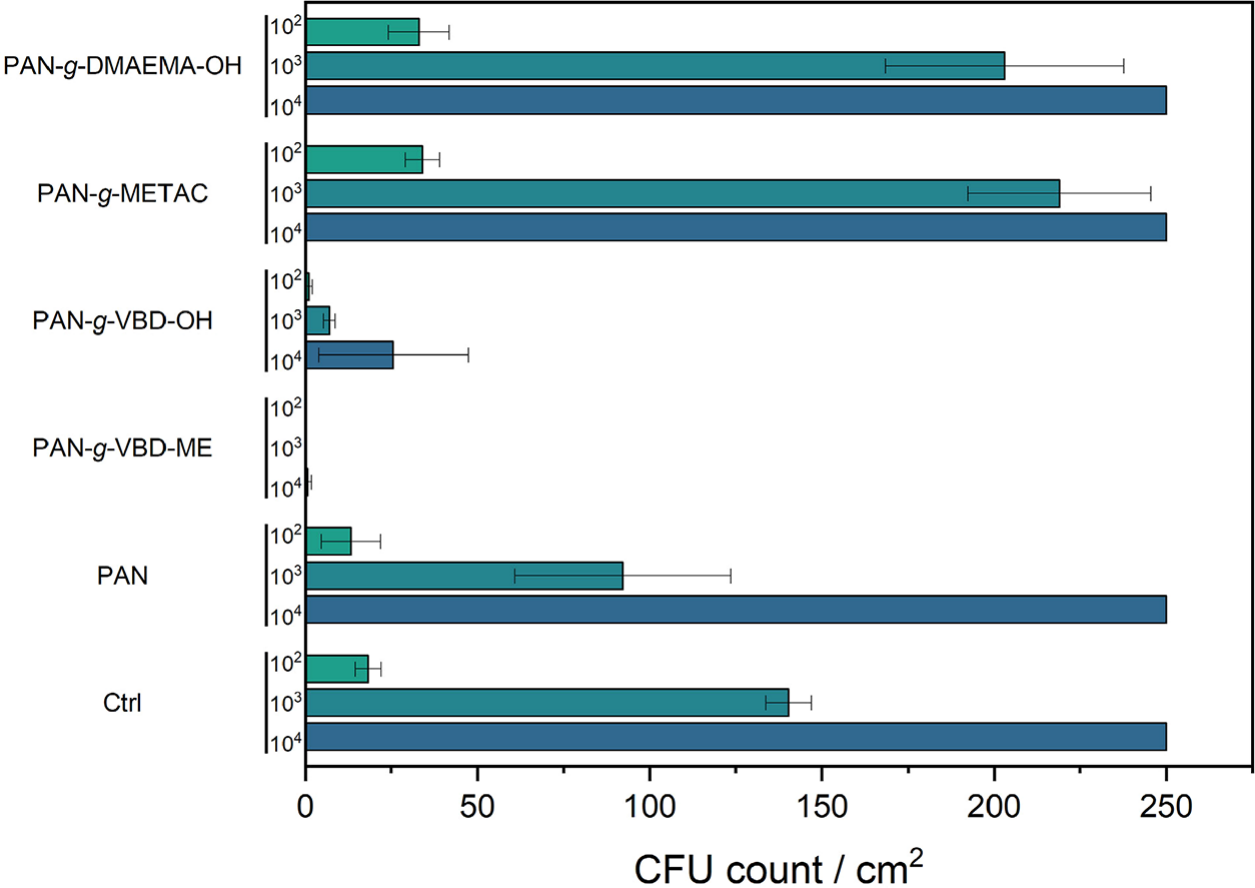
Antibacterial activity of pristine PAN and cationic PAN membranes (DABCO- and DMAEMA-based) were evaluated using a modified ASTM E2149-20 assay with *S. aureus* (ATCC 29213). Membrane samples (1.0 cm^2^) were incubated with 2 mL of bacterial suspension (10^5^ CFU/mL) for 2 h. The suspensions and their serial dilutions (10^4^–10^2^ CFU/mL) were plated on Columbia agar and incubated for 17 h before colony counting. Experiments were performed in triplicate, with pristine PAN (Ctrl) as the control. Colony counts >250 were recorded as too numerous to count.

The microbiological evaluation of the modified membranes revealed that the strongly positively charged PAN-*g*-VBD-ME effectively eliminated all bacteria. A high amount of negative charges (>10^15^ N^+^/cm^2^) is typically assumed to be necessary for a material to have contact-active antimicrobial properties.^65^ This value is hard to determine for porous materials, but the highly positive ζ potential of PAN-*g*-VBD-ME might reflect sufficient charge density. All other materials did not show a significant reduction in bacterial growth, which is supported by the significantly lower values for their surface ζ potential compared to PAN-*g*-VBD-ME. The only exception is PAN-*g*-VBD–OH, which led to a small but significant reduction in bacterial growth. Further studies focusing on bacterial adhesion mechanisms and membrane surface properties are required to elucidate these findings in greater detail.

## Conclusions

Functionalized membranes are valuable tools for water purification. This study introduces a new UV-grafting process of charged and zwitterionic brush polymers to PAN membranes using LAP or BAPO as photoinitiators. Two sets of monomers based on vinylbenzen and methacrylate scaffolds were used for surface grafting. Characterization of the resulting materials through goniometry, IR spectroscopy and ζ potential measurements confirmed the grafting success. A SEM-EDX analysis further validated the successful modification of PAN membranes with the expected functionalized brush polymers and confirmed the presence of the latter on the outer material surface and also in the pores of the membranes. The pore sizes were only slightly affected by the graft-polymerization and the polymerization of methacrylate derivatives led to a higher pore size and porosity compared to the vinylbenzen derivatives. The functionalized membranes retained thus sufficient water flow of around 100 L/(m^2^ h bar).

Adsorption capacity of wastewater contaminants was evaluated with methylene blue and orange II. Several of the modified materials had a high capacity for removal of the negatively charged dye orange II, which correlates well with the observed positive surface ζ potential for example of the cationic PAN-*g*-VBD-ME. The adsorption capacity for the positively charged methylene blue was naturally lower for all materials except pristine PAN. PAN-*g*-VBD-NOx revealed a comparable absorption capacity for both dyes, indicating that electrostatic interactions play a limited role in the adsorption process. Interaction of the styrene backbone with aromatic dyes are most likely important in this context. In addition, PAN-*g*-VBD-ME has a notable antimicrobial activity against *S. aureus*, highlighting its potential for water purification and antifouling. The antimicrobial activity is most likely caused by a high positive charge density on the outer material surface, which is reflected by the strongly positive surface ζ potential of this material.

This study has limitations. The grafting efficiency of the polymer brushes can be significantly different among the monomers tested. Quantitative correlations of the surface ζ potential or the adsorption capacity of charged dyes to the charges of the brush polymer are thus not possible. The adhesion of bacteria to the membrane surface was also not evaluated in this study and will be a subject of future investigation particularly for the membranes modified with zwitterionic brush polymers.

In summary, the UV-grafting of functionalized brush polymers from commercial PAN membranes is a powerful approach to develop functionalized PAN membranes with enhanced performance in water purification and antifouling.

## Supplementary Material



## Notes

Synthetic procedures and analytical data for all new compounds; UV-grafting mechanism (Figures S1 and S2); procedures for UV-grafting on PAN-membranes; ATR-FTIR spectra of all materials (Figures S3 and S4); SEM images of the membrane surfaces and corresponding cross-fractures (Figures S5–S7); AFM images (Figures S8 and S9); EDX-spectra and line spectrum of sulfur within the cross-fracture of PAN-*g*-DMAEMA-SB and PAN-*g*-VBD-SB (Figures S10–S13); values for pore size, porosity, WCA, roughness and MWCO for pristine PAN and all modified PAN membranes (Table S1); additional microbiological data derived from the ASTM E2149-20 assay (Figure S14); ^1^H and ^13^C NMR spectra of VBD, VBD-SB, VBD-ME and pDMAEMA (synthesized in solution) (Figures S15–S23); ESI-MS-spectra of VBD, VBD-SB and VBD-ME (Figures S24–S27) (PDF)

## Abbreviations

BAPO: phenylbis­(2,4,6-trimethylbenzoyl)­phosphine oxide
BSA: bovine serum albumin
CB: carboxybetaine
CFU: colony forming units
DABCO: 1,4-diazabicyclo­[2.2.2]­octane
DMAEMA: 2-(dimethylamino)­ethyl methacrylate
LAP: lithium phenyl­(2,4,6-trimethylbenzoyl)­phosphinate
METAC: [2-(methacryloyloxy)­ethyl]­trimethylammonium chloride
MWCO: molecular weight cutoff
MPC: 2-methacryloyloxyethyl phosphorylcholine
NIPS: nonsolvent induced phase separation
NOx: *N*-oxide
PAN: polyacrylonitril
PBS: phosphate-buffered saline
PEG: polyethylene glycol
SB: sulfobetaine
UV: ultraviolet
VBD: 1-(4-vinylbenzyl)-1,4-diazabicyclo­[2.2.2]­octan-1-ium chloride
VBD-SB: 3-(4-(4-vinylbenzyl)-1,4-diazabicyclo­[2.2.2]­octan-1,4-diium-1-yl)­propane-1-sulfonate chloride
WCA: water contact angle
