# Supplementary material for: Postfunctionalization of PAN Membranes via UV-Grafting of Charged and Zwitterionic Polymer Brushes
Source: Langmuir. 2025 Dec 22;42(1):949–61. doi: 10.1021/acs.langmuir.5c05068 (PMC12810372; doi:10.1021/acs.langmuir.5c05068)
Supplement: Supplementary file 1 [file la5c05068_si_001.pdf]

Supporting Information

Post-functionalization of PAN membranes *via* UV-grafting of charged and zwitterionic polymer brushes

Timo Friedrich,[a] Donovan Timm,[a] Sarah Glass,[b] Erik S. Schneider,[b] Volkan Filiz[b] and Wolfgang Maison[a,*]

[a]Universität Hamburg, Department of Chemistry, Bundesstrasse 45, 20146 Hamburg, Germany

[b]Institute of Membrane Research, Helmholtz-Zentrum Hereon, Max-Planck-Str. 1, 21502 Geesthacht, Germany

* corresponding author: Wolfgang Maison. Email: wolfgang.maison@uni-hamburg.de

Number of pages: 27

Number of figures: 27

Number of tables: 1

Table of Contents

1) Synthesis 3
2) UV-grafting mechanism for acylphosphine oxides (e.g. BAPO or LAP) 5
3) UV-grafting of on PAN-Membranes...6
4) Post-functionalization of PAN-Membranes 8
5) ATR-FTIR spectroscopy...10
6) Scanning electron microscopy (SEM)..11
7) Atomic force microscopy (AFM)...14
8) Energy-dispersive X-ray spectroscopy (EDX)..17
9) Pore size, Porosity, WCA, Roughness, Molecular weight cut-off 19
10) Antibacterial assay 20
11) NMR-Spectra 21
12) HRMS-Spectra:...26
13) Literature..28

1) Synthesis

1-(4-Vinylbenzyl)-1,4-diazabicyclo[2.2.2]octan-1-ium chloride (VBD)

The title compound was synthesized according to a modified literature procedure from BURMEISTER *et al.*.[1] 1,4-Diazabicyclo[2.2.2]octane (1.25 g, 8.19 mmol, 1.1 eq.) was dissolved in ethyl acetate (50 mL) and cooled to 0 °C. 4-Vinylbenzyl chloride (1.2 mL, 8.19 mmol, 1.0 eq.) was added dropwise. The solution was stirred at room temperature for 16 h. Filtration of the suspension, washing with ethyl acetate (250 mL) and drying in vacuo gave the title compound (1.99 g, 7.52 mmol, 92%) as a light orange solid.

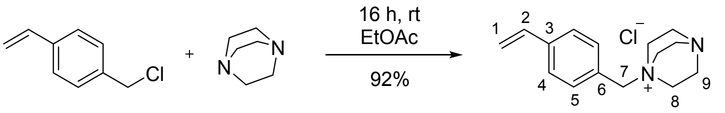

1H-NMR: (600 MHz, D_2O, 25 °C) δ [ppm] = 7.62 (d, $^3J_{HH}$ = 8.2 Hz, 2H, **4-H**), 7.47 (d, $^3J_{HH}$ = 8.2 Hz, 2H, **5-H**), 6.84 (dd, $^3J_{HH}$ = 17.7, 11.0 Hz, 1H, **2-H**), 5.95 (d, $^3J_{HH}$ = 18.5 Hz, 1H, **1a-H**), 5.43 (d, $^3J_{HH}$ = 11.7 Hz, 1H, **1b-H**), 4.47 (s, 2H, **7-H**), 3.44 (t, $^3J_{HH}$ = 7.88 Hz, 6H, **8-H**), 3.17 (t, $^3J_{HH}$ = 7.63, 6H, **9-H**). **^{13}C-NMR:** (150 MHz, D_2O, 25 °C) δ [ppm] = 139.71 (**C3**), 135.69 (**C2**), 133.3 (**C5**), 126.7 (**C4**), 125.3 (**C6**), 116.3 (**C1**), 68.0 (**C7**), 52.0 (**C8**), 44.2 (**C9**). **HRMS** (ESI^+) m/z: $[M]^+$ calculated, 229.1699; found, 229.1691.

3-(4-(4-Vinylbenzyl)-1,4-diazabicyclo[2.2.2]octan-1,4-diium-1-yl)propane-1-sulfonate 3-hydroxypropane-1-sulfonate chloride (VBD-SB)

The title compound was synthesized according to a literature procedure from BURMEISTER *et al.*.[1] 1-(4-Vinylbenzyl)-1,4-diazabicyclo[2.2.2]octan-1-ium chloride (5.0 g, 18.9 mmol, 1.0 eq.) was dissolved in MeCN (50 mL) and 1,3-propane sultone (3.3 mL, 37.8 mmol, 2.0 eq.) was added dropwise. The solution was stirred at room temperature for 16 h. Filtration of the suspension, washing with MeCN (250 mL) and drying *in vacuo* gave the title compound as a colorless solid (4,8 g, 12,4 mmol, 65%).

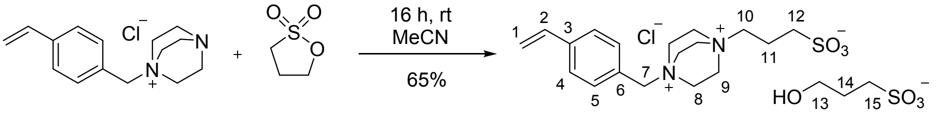

1H-NMR: (600 MHz, D_2O, 25 °C) δ [ppm] = 7.68 (d, $^3J_{HH}$ = 8.2 Hz, 2H, **4-H**), 7.55 (d, $^3J_{HH}$ = 8.3 Hz, 2H, **5-H**), 6.86 (dd, $^3J_{HH}$ = 17.7, 11.0 Hz, 1H, **2-H**), 5.99 (d, $^3J_{HH}$ = 17.0 Hz, 1H, **1a-H**), 5.46 (d, $^3J_{HH}$ = 11.0 Hz, 1H, **1b-H**), 4.80 (s, 3H, **7-H**), 4.03 (s, 12H, **8-H**, **9-H**), 3.78 – 3.71 (m, 4H, **10-H**, **13-H**), 3.09 – 3.04 (m, 2H, **12-H**), 3.01 (t, $^3J_{HH}$ = 7.1 Hz, 2H, **15-H**), 2.32 – 2.24 (m, 2H, **11-H**), 2.24 – 2.17 (m, 2H, **14-H**). **^{13}C-NMR:** (150 MHz, D_2O, 25 °C) δ [ppm] = 140.5

(**C3**), 135.6 (**C2**), 133.2 (**C5**), 127.2 (**C4**), 124.0 (**C6**), 116.8 (**C1**), 68.6 (**C7**), 63.3 (**C10**), 51.3 (**C8**), 50.7 (**C9**), 48.3 (**C12**), 46.8 (**C15**), 43.5 (**C13**), 27.4 (**C11**), 17.7 (**C14**). **HRMS** (ESI^+) m/z: $[M]^+$ calculated, 351.1731; found, 351.1741.

1-Methyl-4-(4-vinylbenzyl)-1,4-diazabicyclo[2.2.2]octane-1,4-diium chloride iodide (VBD-ME)

1-(4-Vinylbenzyl)-1,4-diazabicyclo[2.2.2]octan-1-ium chloride (5.0 g, 18.9 mmol, 1.0 eq.) was dissolved in MeCN (50 mL) and methyl iodide (1.3 mL, 20.8 mmol, 1.1 eq.) was added dropwise. The solution was stirred at room temperature for 16 h. Filtration of the suspension, washing with MeCN (250 mL) and drying *in vacuo* gave the title compound as a colorless solid (3.14 g, 7.71 mmol, 41%).

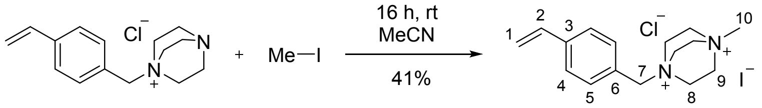

^{1}H-NMR (600 MHz, D_2O) δ = 7.68 (d, $^3J_{HH}$ = 8.2 Hz, 2H, **4-H**), 7.55 (d, $^3J_{HH}$ = 8.2 Hz, 2H, **5-H**), 6.87 (dd, $^3J_{HH}$ = 17.7, 10.9 Hz, 1H, **2-H**), 5.99 (d, $^3J_{HH}$ = 18.4 Hz, 1H, **1a-H**), 5.46 (d, $^3J_{HH}$ = 11.6 Hz, 1H, **1b-H**), 4.82 (s, 2H, **7-H**), 4.05 (s, 12H, **8-H**, **9-H**), 3.39 (s, 3H, **10-H**). **^{13}C-NMR:** (150 MHz, D_2O, 25 °C) δ [ppm] = 140.5 (**C3**), 135.6 (**C2**), 133.3 (**C5**), 127.2 (**C4**), 124.0 (**C6**), 116.8 (**C1**), 68.7 (**C7**), 53.4 (**C8**), 52.5 (**C10**), 50.7 (**C9**). **HRMS** (ESI^+) m/z: $[M]^{2+}$ calculated, 122.0964; found, 122.0949.

2) UV-grafting mechanism for acylphosphine oxides (e.g. BAPO or LAP)

BAPO and LAP are acylphosphine oxide photoinitiators that generate radicals through homolytic bond cleavage upon UV-Vis irradiation, typically around 365 nm. After light absorption, the molecule is excited to a singlet state and can undergo intersystem crossing to the triplet state. From this excited triplet state, Norrish Type I α-cleavage occurs at the carbon–phosphorus bond adjacent to the carbonyl group, leading to the formation of an acyl radical and a phosphinoyl radical. Both species can initiate polymerization.[2, 3] Due to the presence of two acyl groups, BAPO can produce up to four radicals per molecule, resulting in higher photoinitiation efficiency compared to monoacylphosphine oxides such as LAP.

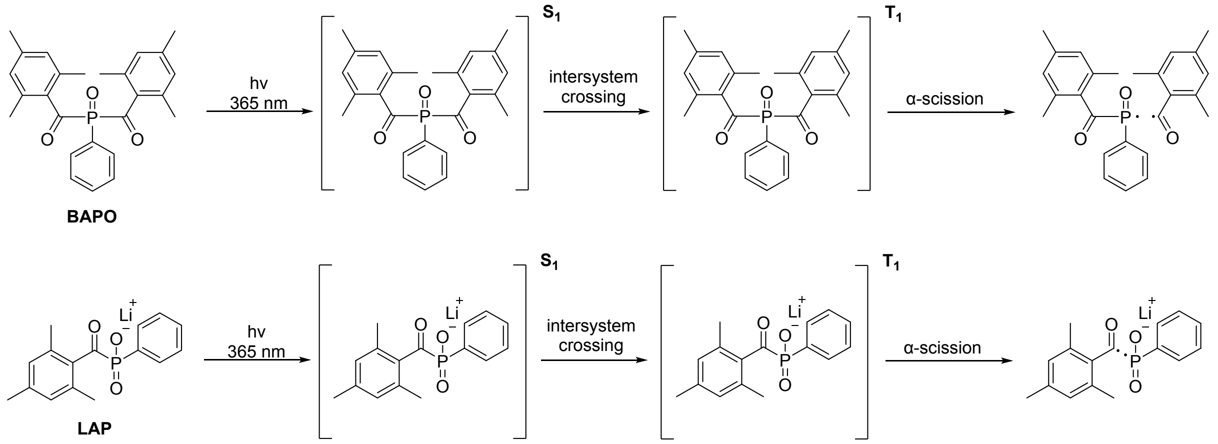

Figure S1: Mechanism of radical formation for BAPO and LAP.

The radical species formed in the photolysis of LAP and BAPO can initiate a graft polymerization on the PAN surface. The generated radicals are capable of abstracting a hydrogen atom from the PAN backbone, thereby creating a surface-bound radical site. This surface radical serves as the initiation point for the graft polymerization. Subsequently, the radical can react with monomers such as methacrylate or styrene derivatives, leading to the growth of polymer chains from the surface and the formation of a polymer brush structure.

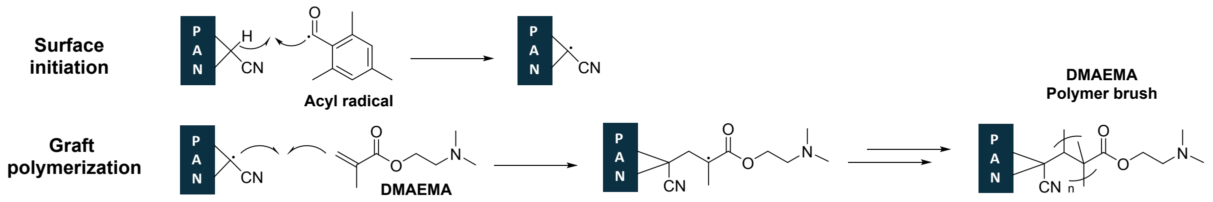

Figure S2: Proposed mechanism of graft polymerization initiated by the acyl radical using DMAEMA as the monomer.

3) UV-grafting of on PAN-Membranes

1-(4-Vinylbenzyl)-1,4-diazabicyclo[2.2.2]octan-1-ium chloride (PAN-*g*-VBD)

1-(4-Vinylbenzyl)-1,4-diazabicyclo[2.2.2]octan-1-ium chloride (0.50 g, 1.89 mmol, 1.0 eq.) and LAP (0.011 g, 0.038 mmol, 2.0 mol%) were dissolved in demineralized water (2 mL). PAN membrane with an area of 1.0 cm^2 was added to the solution and was degassed with nitrogen for 20 min. The soaked PAN membrane was transferred to a new vial and was irradiated with UV light (365 nm, 30 W) for 2 h at room temperature. The modified PAN membrane was subsequently cleaned with demineralized water (10 mL) in an ultrasonic bath three times 10 min and dried *in vacuo* at 50 °C.

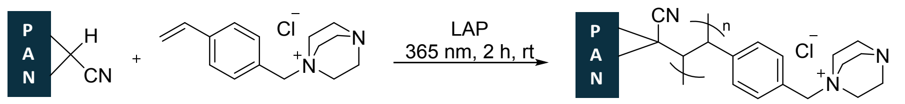

3-(4-(4-Vinylbenzyl)-1,4-diazabicyclo[2.2.2]octan-1,4-diium-1-yl)propane-1-sulfonate 3-hydroxypropane-1-sulfonate (PAN-*g*-VBD-SB)

3-(4-(4-Vinylbenzyl)-1,4-diazabicyclo[2.2.2]octan-1,4-diium-1-yl)propane-1-sulfonate 3-hydroxypropane-1-sulfonate (0.50 g, 1.02 mmol, 1.0 eq.) and LAP (0.006 g, 0.02 mmol, 2 mol%) were dissolved in demineralized water (2 mL). PAN membrane with an area of 1.0 cm^2 was added to the solution and was degassed with nitrogen for 20 min. The soaked PAN membrane was transferred to a new vial and was irradiated with UV light (365 nm, 30 W) for 2 h at room temperature. The modified PAN membrane was subsequently cleaned with demineralized water (10 mL) in an ultrasonic bath three times 10 min and dried *in vacuo* at 50 °C.

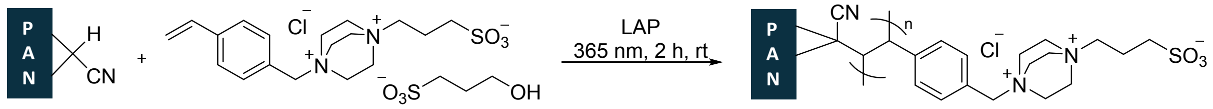

1-Methyl-4-(4-vinylbenzyl)-1,4-diazabicyclo[2.2.2]octane-1,4-diium chloride iodide (PAN-*g*-VBD-ME)

1-Methyl-4-(4-vinylbenzyl)-1,4-diazabicyclo[2.2.2]octane-1,4-diium chloride iodide (0.50 g, 1.23 mmol, 1.0 eq.) and LAP (0.007 g, 0.03 mmol, 2 mol%) were dissolved in demineralized water (2 mL). PAN membrane with an area of 1.0 cm^2 was added to the solution and was degassed with nitrogen for 20 min. The soaked PAN membrane was transferred to a new vial and was irradiated with UV light (365 nm, 30 W) for 2 h at room temperature. The modified PAN membrane was subsequently cleaned with demineralized water (10 mL) in an ultrasonic bath three times 10 min and dried *in vacuo* at 50 °C.

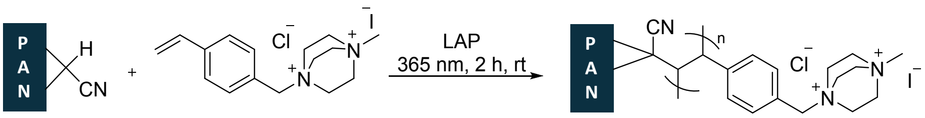

2-(Dimethylamino)ethyl methacrylate (PAN-*g*-DMAEMA)

2-(Dimethylamino)ethyl methacrylate (5.00 mL, 29.8 mmol, 1.0 eq.) and BAPO (0.125 g, 0.298 mmol, 1 mol%) were dissolved. PAN membrane with an area of 1.0 cm^2 was added to the solution and was degassed with nitrogen for 20 min. The soaked PAN membrane was transferred to a new vial and was irradiated with UV light (365 nm, 30 W) for 1 h at room temperature. The modified PAN membrane was subsequently cleaned with demineralized water (10 mL) in an ultrasonic bath three times 10 min and dried *in vacuo* at 50 °C.

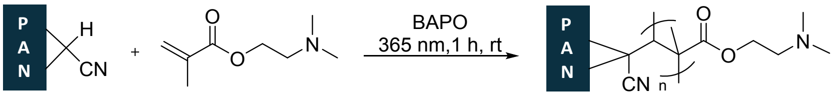

2-(Methacryloyloxy)ethyl]trimethylammonium chloride (PAN-*g*-METAC)

2-(Methacryloyloxy)ethyl]trimethylammonium chloride (75% in H_2O, 1.30 mL, 3.38 mmol, 1.0 eq.) and LAP (0.030 g, 0.103 mmol, 2.0 mol%) were dissolved in demineralized water 4 mL). PAN membrane with an area of 1.0 cm^2 was added to the solution and was degassed with nitrogen for 20 min. The soaked PAN membrane was transferred to a new vial and was irradiated with UV light (365 nm, 30 W) for 1 h at room temperature. The modified PAN membrane was subsequently cleaned with demineralized water (10 mL) in an ultrasonic bath three times 10 min and dried *in vacuo* at 50 °C.

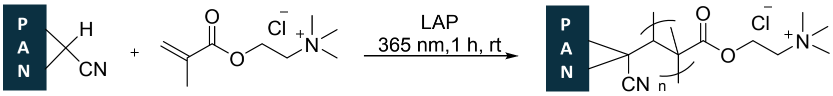

2-Methacryloyloxyethyl phosphorylcholine (PAN-*g*-MPC)

2-Methacryloyloxyethyl phosphorylcholine (1.00 g, 3.38 mmol, 1.0 eq.) and LAP (0.020 g, 0.068 mmol, 2.0 mol%) were dissolved in 4 mL demineralized water. PAN membrane with an area of 1.0 cm^2 was added to the solution and was degassed with nitrogen for 20 min. The soaked PAN membrane was transferred to a new vial and was irradiated with UV light (365 nm, 30 W) for 1 h at room temperature. The modified PAN membrane was subsequently cleaned with demineralized water (10 mL) in an ultrasonic bath three times 10 min and dried *in vacuo* at 50 °C.

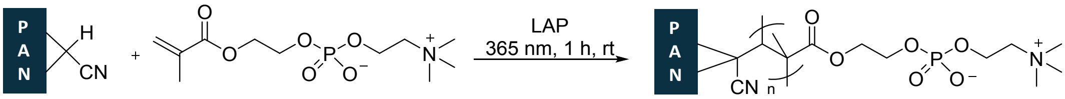

4) Post-functionalization of PAN-Membranes

PAN-*g*-VBD functionalization with H_2O_2 (PAN-*g*-VBD-NOx)

PAN-*g*-VBD membrane with an area of 1.0 cm^2 was oxidized with H_2O_2 (2 mL, 30% w/w) for 24 h at 50 °C. The membrane was washed with deionized water and was shaken in a thiosulfate solution (2 mL, 1 M) for 30 min to quench the excess of H_2O_2. The modified PAN membrane was subsequently cleaned with deionized water (10 mL) in an ultrasonic bath three times 10 min and dried *in vacuo* at 50 °C.

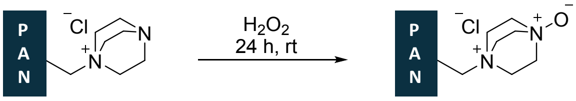

PAN-*g*-VBD functionalization with sodium chloroacetate (PAN-*g*-VBD-CB)

PAN-p-VBD membrane with an area of 1.0 cm^2 was alkylated with chloroacetate solution (2 mL, 1 M) for 24 h at 50 °C. The modified PAN membrane was subsequently cleaned with deionized water (10 mL) in an ultrasonic bath three times 10 min and dried *in vacuo* at 50 °C.

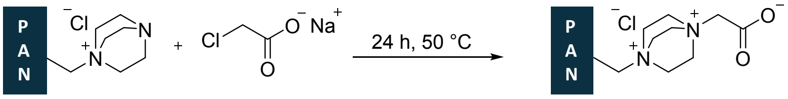

PAN-*g*-VBD functionalization with 2-bromoethanol (PAN-*g*-VBD-OH)

PAN-p-VBD membrane with an area of 1.0 cm^2 was alkylated with 2-bromoethanol (2.0 mL, 28.2 mmol) for 24 h at 50 °C. The modified PAN membrane was subsequently cleaned with ethanol (10 mL) in an ultrasonic bath three times 10 min and dried *in vacuo* at 50 °C.

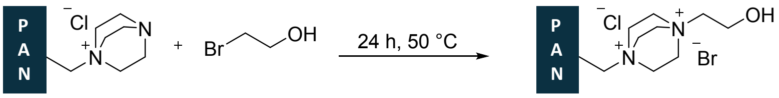

PAN-*g*-DMAEMA functionalization with H_2O_2 (PAN-*g*-DMAEMA-NOx)

PAN-p-DMAEMA membrane with an area of 1.0 cm^2 was oxidized with H_2O_2 (2 mL, 30% w/w) for 24 h at 50 °C. The membrane was washed with deionized water and was shaken in a thiosulfate solution (2 mL, 1 M) for 30 min to quench the excess of H_2O_2. The modified PAN membrane was subsequently cleaned with deionized water (10 mL) in an ultrasonic bath three times 10 min and dried *in vacuo* at 50 °C.

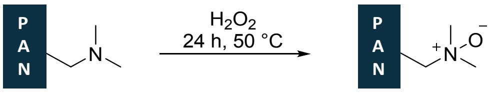

PAN-*g*-DMAEMA functionalization with sodium chloroacetate (PAN-*g*-DMAEMA-CB)

PAN-p-DMAEMA membrane with an area of 1.0 cm^2 was alkylated with chloroacetate solution (2 mL, 1 M) for 24 h at 50 °C. The modified PAN membrane was subsequently cleaned with deionized water (10 mL) in an ultrasonic bath three times 10 min and dried *in vacuo* at 50 °C.

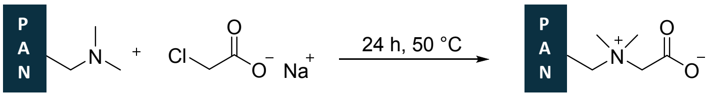

PAN-*g*-DMAEMA functionalization with sodium 2-bromoethanesulfonate (PAN-*g*-DMAEMA-SB)

PAN-pDMAEMA membrane with an area of 1.0 cm^2 was alkylated with 2-bromoethanolsulfonate solution (2 mL, 1 M) for 24 h at 50 °C. The modified PAN membrane was subsequently cleaned with deionized water (10 mL) in an ultrasonic bath three times 10 min and dried *in vacuo* at 50 °C.

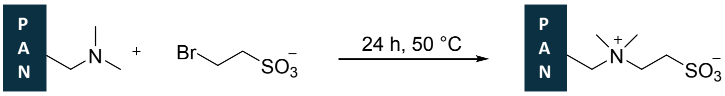

PAN-*g*-DMAEMA functionalization with 2-bromoethanol (PAN-*g*-DMAEMA-OH)

PAN-p-DMAEMA membrane with an area of 1.0 cm^2 was alkylated with 2-bromoethanol (2.0 mL, 28.2 mmol) for 24 h at 50 °C. The modified PAN membrane was subsequently cleaned with ethanol (10 mL) in an ultrasonic bath three times 10 min and dried *in vacuo* at 50 °C.

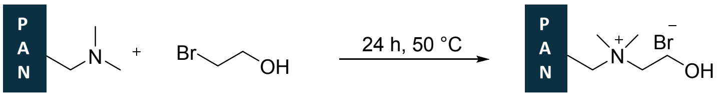

5) ATR-FTIR spectroscopy

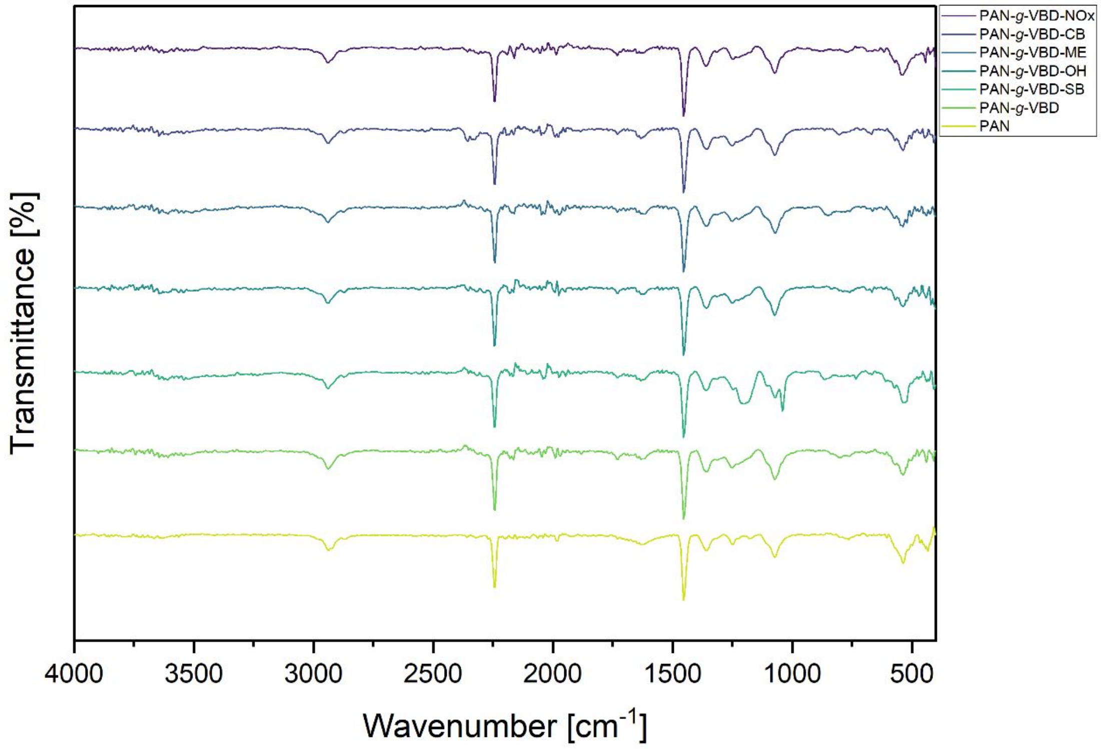

Figure S3: ATR-FTIR spectra in transmisson mode of pristine PAN, PAN-*g*-VBD-CB, PAN-*g*-VBD-SB, PAN-*g*-VBD-OH, PAN-*g*-VBD-ME, PAN-*g*-VBD-CB and PAN-*g*-VBD-NOx.

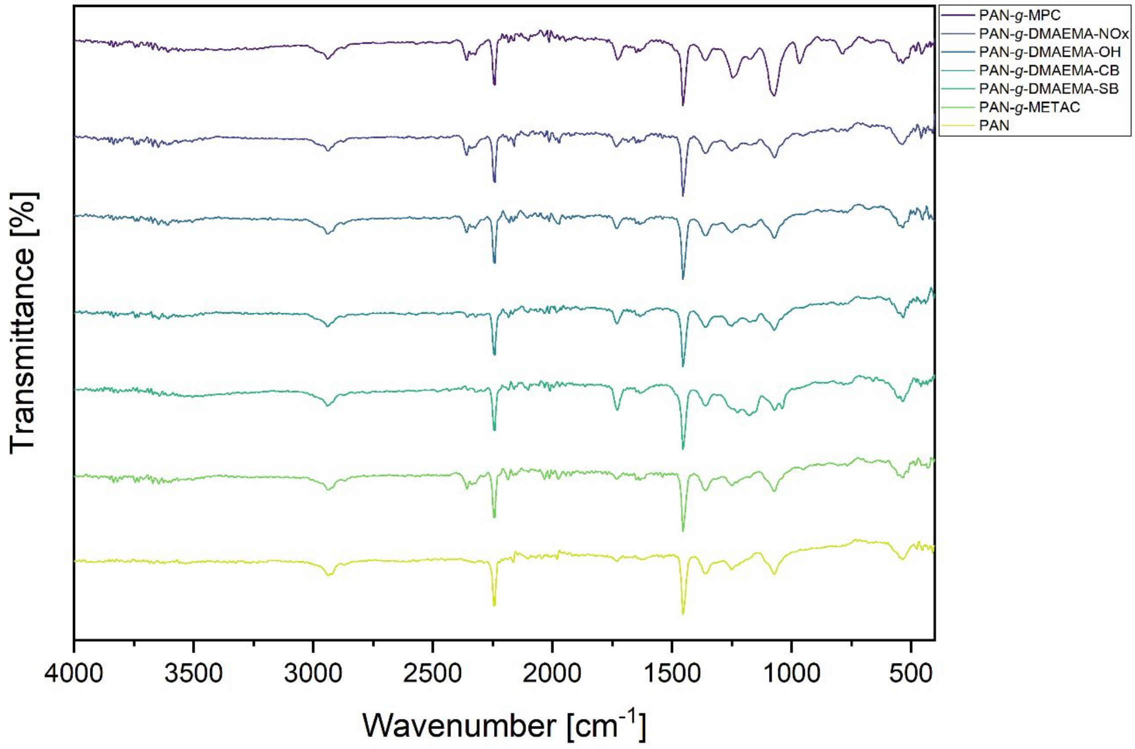

Figure S4: ATR-FTIR spectra in transmission mode of pristine PAN, PAN-*g*-METAC, PAN-*g*-DMAEMA-SB, PAN-*g*-DMAEMA-CB, PAN-*g*-DMAEMA-OH, PAN-*g*-DMAEMA-NOx and PAN-*g*-MPC.

6) Scanning electron microscopy (SEM)

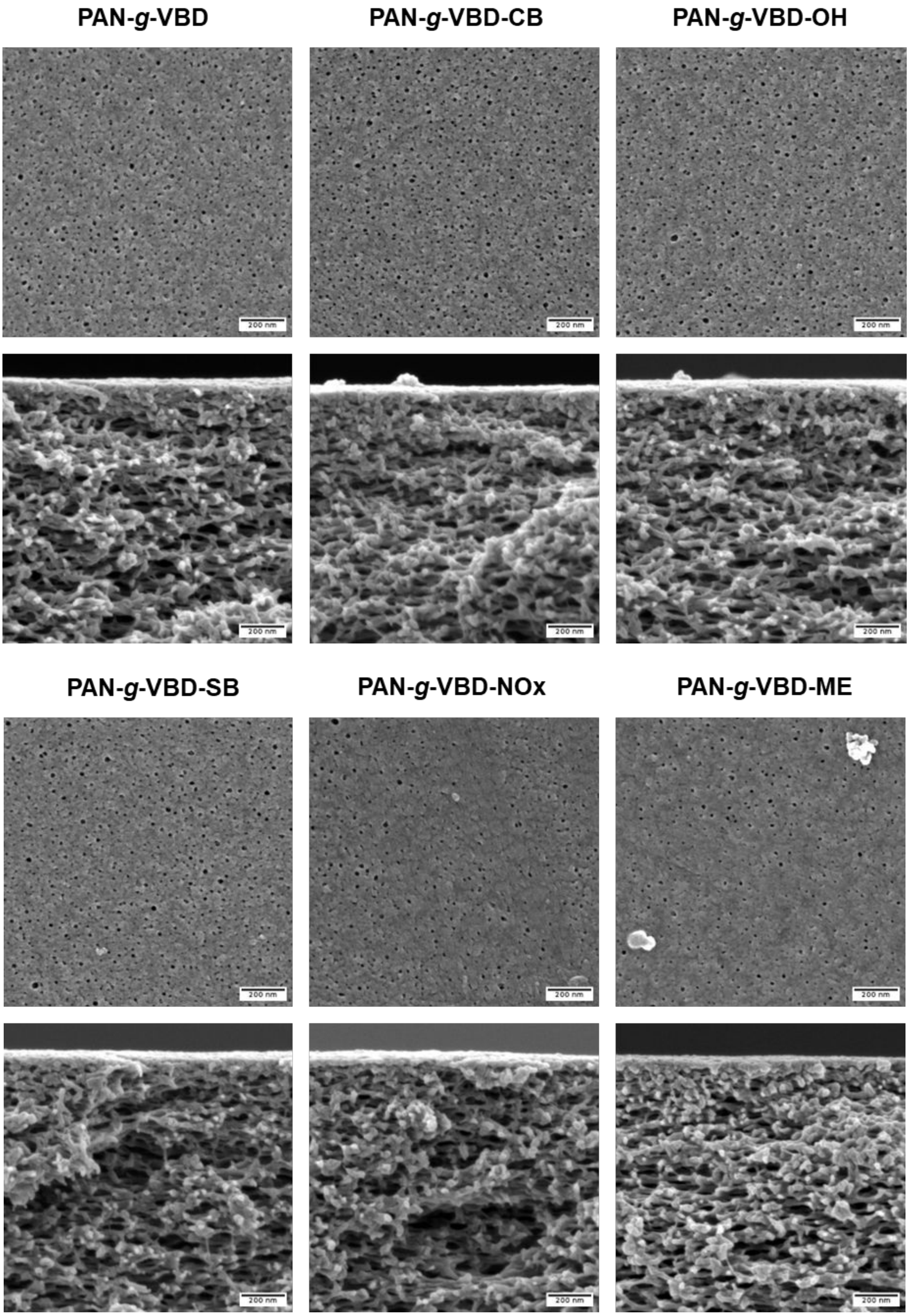

Figure S5: SEM images of the surface in a 50k magnification of modified PAN membranes with VBD, VBD-CB, VBD-OH, VBD-SB, VBD-NOx and VBD-ME and SEM images of the corresponding cross-fractures of modified PAN membranes.

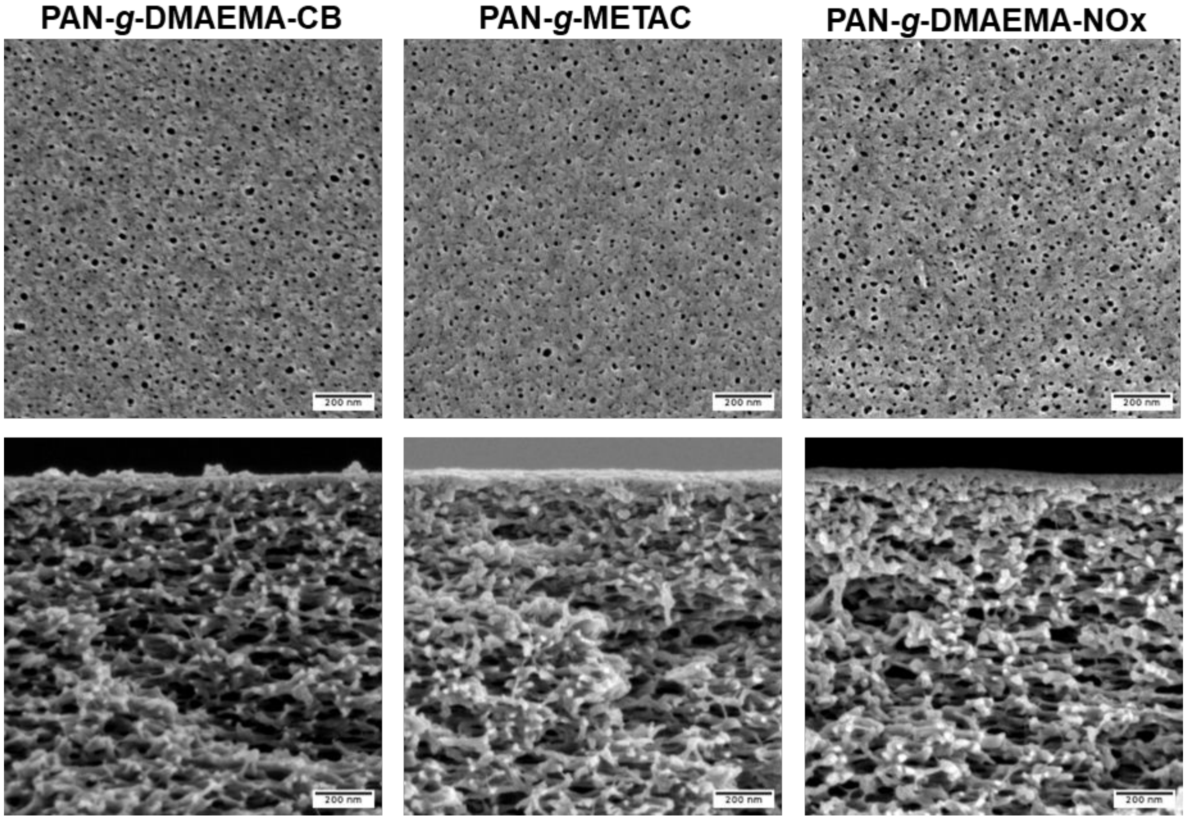

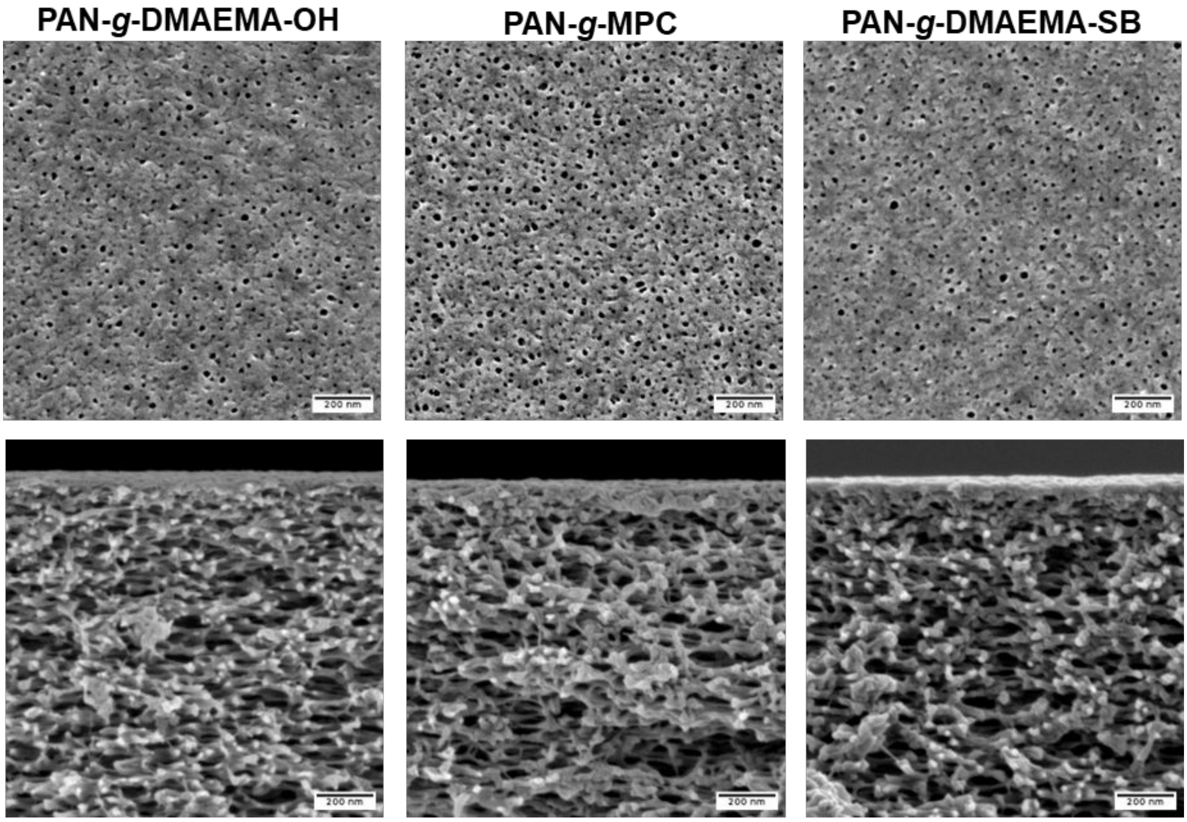

Figure S6: SEM images of the surface in a 50k magnification of modified PAN membranes with DMAEMA-CB, METAC, DMAEMA-NOx, DMAEMA-OH, MPC and DMAEMA-SB and SEM images of the corresponding cross-fractures of modified PAN membranes.

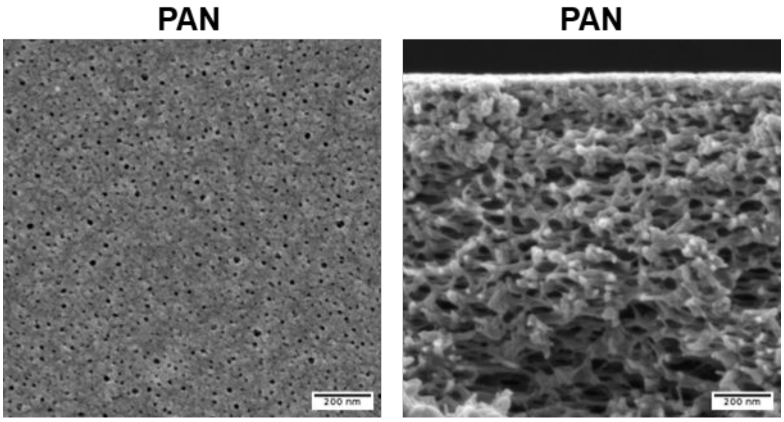

Figure S7: SEM images of the surface in a 50k magnification of pristine PAN membranes and SEM images of the corresponding cross-fracture.

7) Atomic force microscopy (AFM)

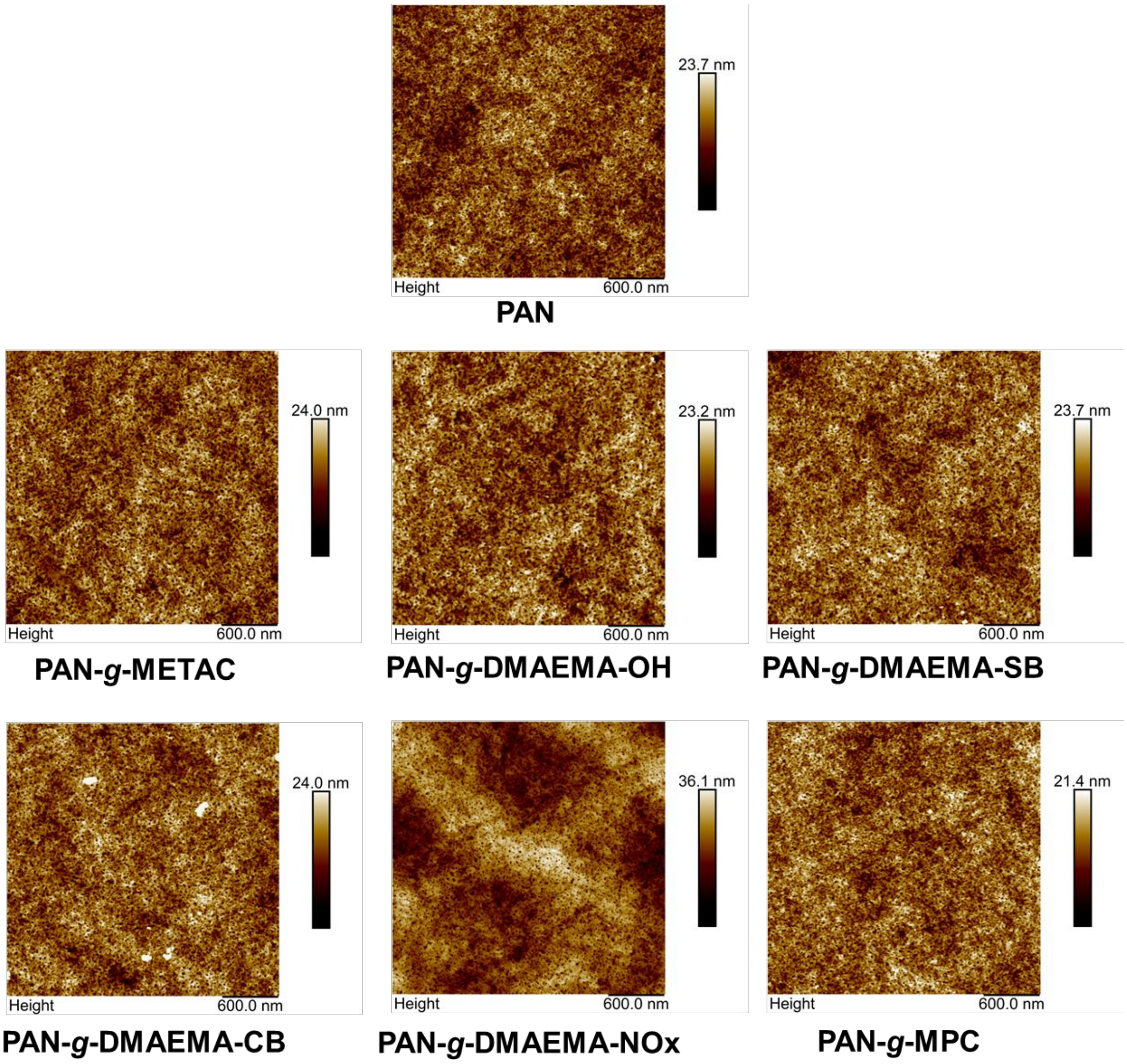

Figure S8: AFM images of the surface of modified PAN membranes with DMAEMA-CB, METAC, DMAEMA-NOx, DMAEMA-OH, MPC and DMAEMA-SB.

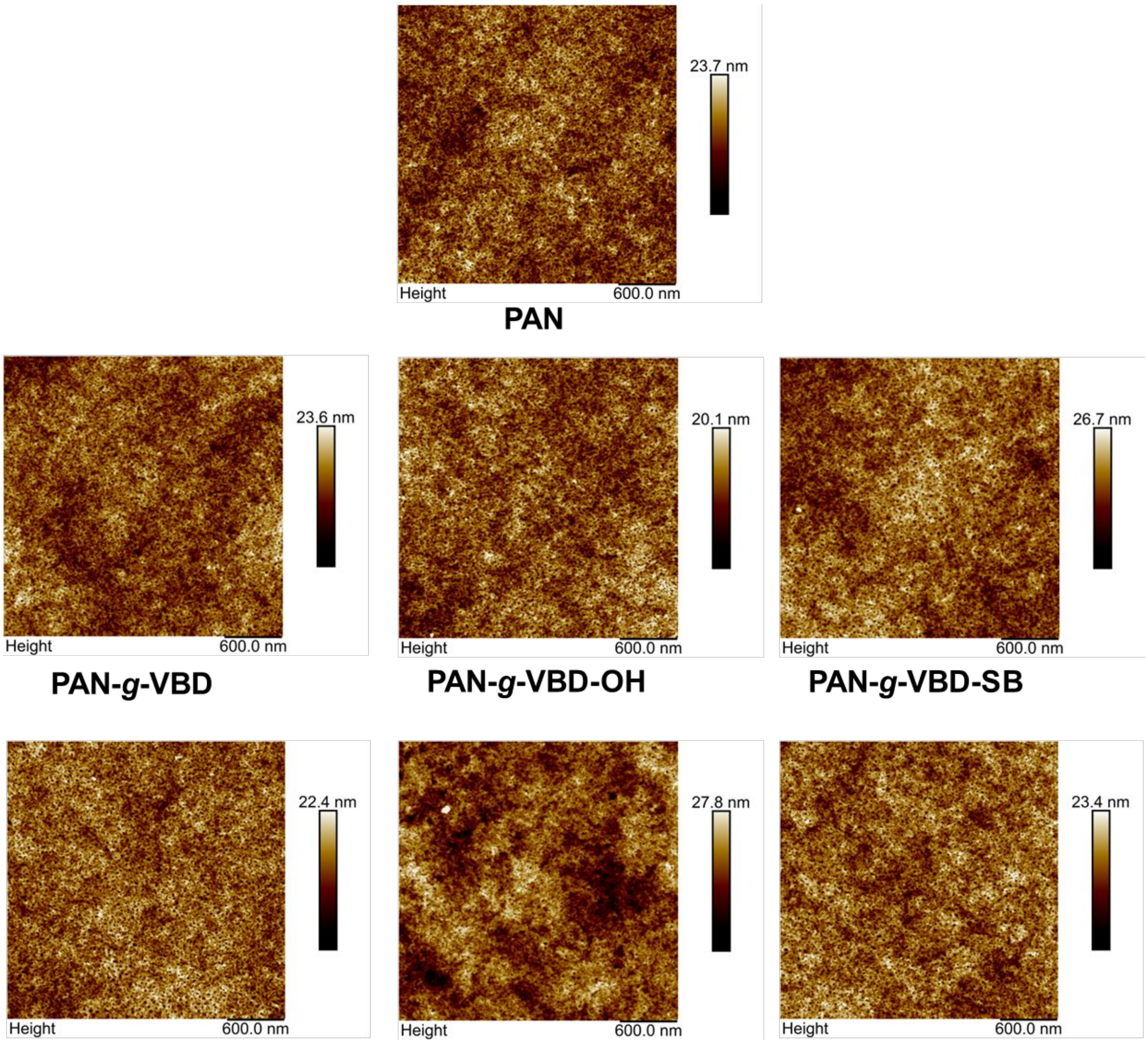

Figure S9: AFM images of the surface of modified PAN membranes with VBD, VBD-CB, VBD-OH, VBD-SB, VBD-NOx and VBD-ME.

8) Energy-dispersive X-ray spectroscopy (EDX)

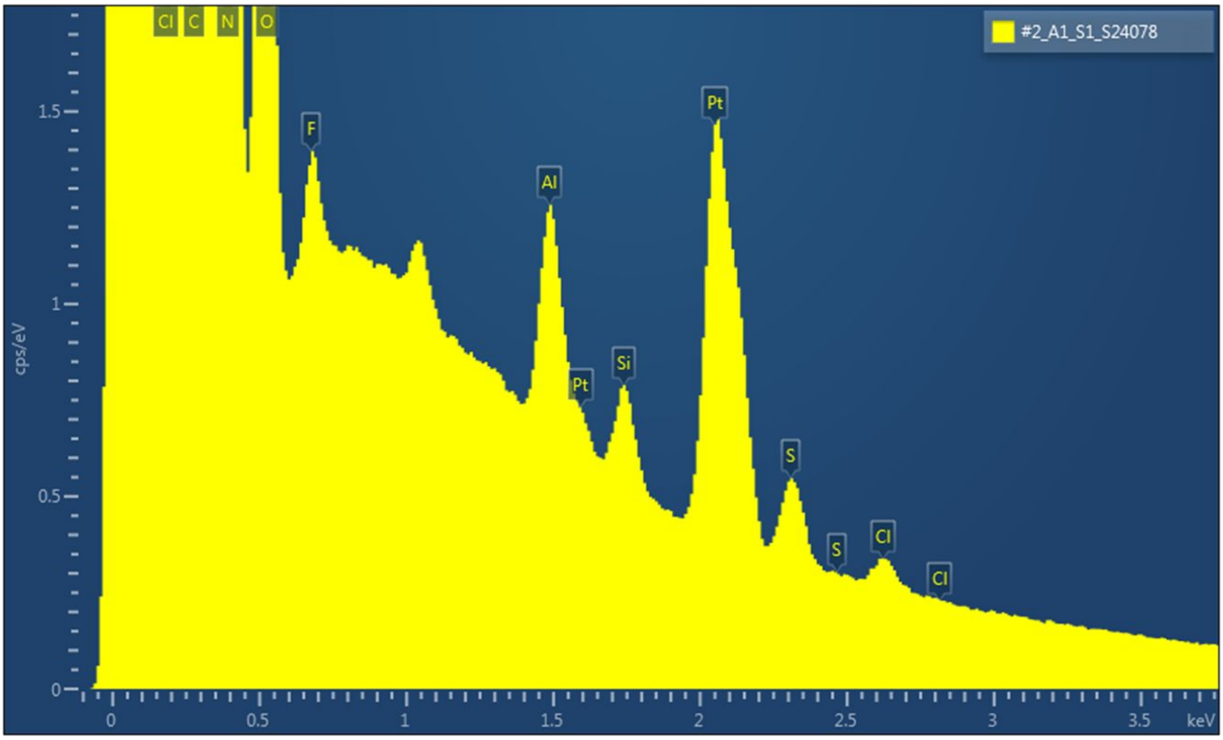

Figure S10: Zoomed-in EDX-spectra of the surface of PAN modified with DMAEMA-SB.

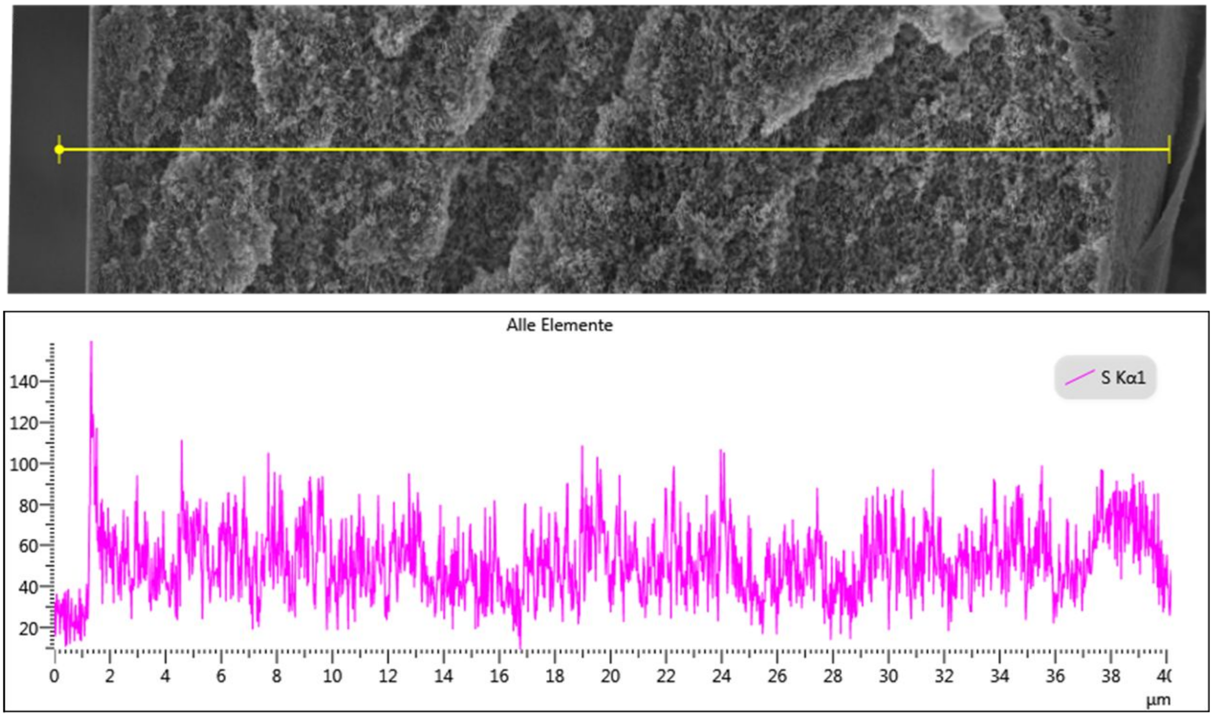

Figure S11: SEM image of the cross-fracture and line spectrum of sulfur within the cross-fracture of PAN modified with DMAEMA-SB.

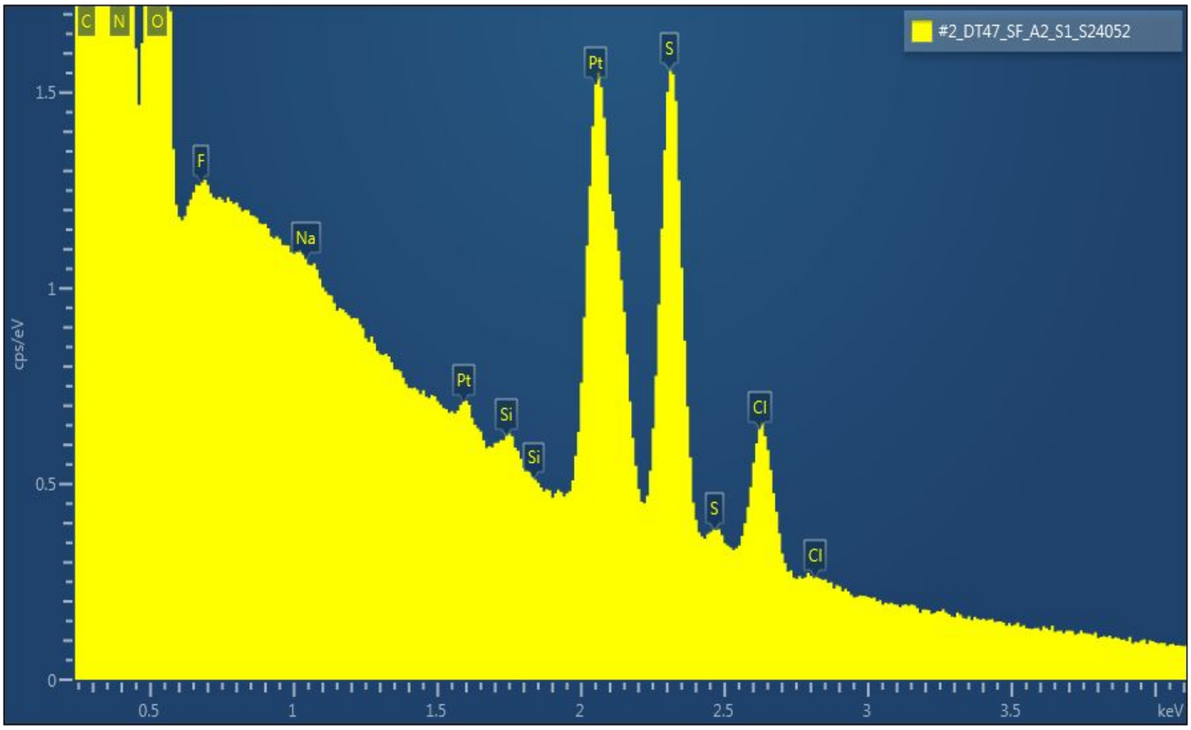

Figure S12: Zoomed in EDX-spectra of the surface of PAN modified with VBD-SB.

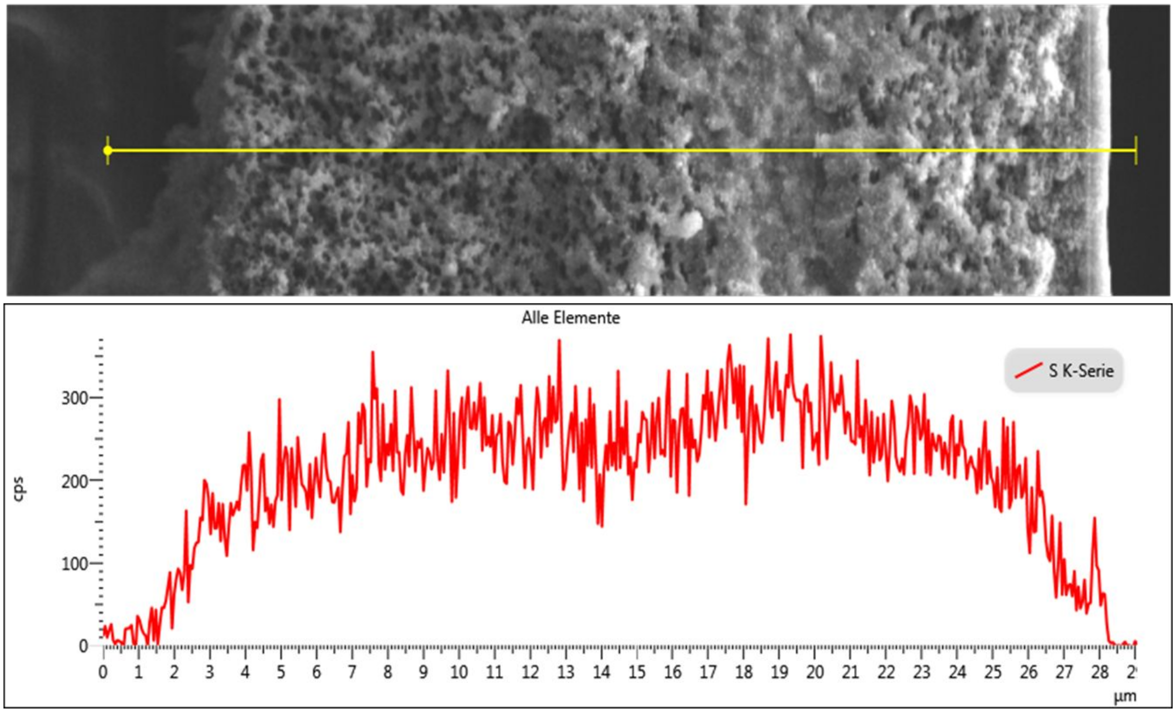

Figure S13: SEM image of the cross-fracture and line spectrum of sulfur within the cross-fracture of PAN modified with VBD-SB.

9) Pore size, Porosity, WCA, Roughness, Molecular weight cut-off

Table S1: Key characteristics of PAN and its modifications, including pore size, porosity, water contact angle (WCA), roughness, and molecular weight cut-off.

**PAN modifications**	**Pore size [nm]**	**Porosity [%]**	**Water contact angle [°]**	**Roughness R_a [nm]**	**Molecular weight cut-off [kDa]**
PAN	8.4	1.79	44.8	3.38	351
PAN-*g*-VBD	8.3	1.44	48.5	2.54	351
PAN-*g*-VBD-ME	8.5	0.88	42.4	3.29	279
PAN-*g*-VBD-SB	8.4	1.82	47.8	2.41	351
PAN-*g*-VBD-CB	8.2	1.85	43.5	2.32	279
PAN-*g*-VBD-NOx	8.4	1.31	34.3	4.01	351
PAN-*g*-VBD-OH	8.1	1.73	39.5	2.44	351
PAN-*g*-METAC	8.9	2.31	44.7	2.70	351
PAN-*g*-DMAEMA-OH	9.2	2.23	61.0	2.76	351
PAN-*g*-DMAEMA-NOx	10.5	5.04	46.0	3.71	279
PAN-*g*-DMAEMA-SB	9.8	1.65	52.2	2.56	279
PAN-*g*-DMAEMA-CB	10.4	3.36	55.9	2.81	279
PAN-*g*-MPC	11.1	5.40	45.7	2.34	279

10) Antibacterial assay

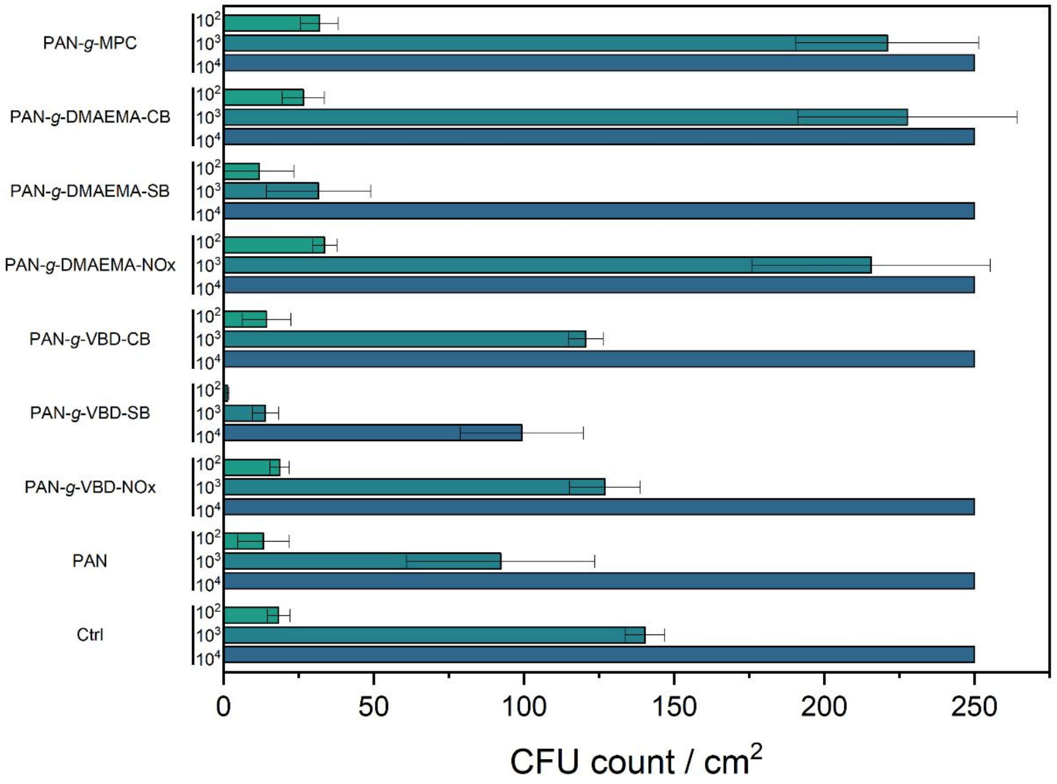

Figure S14: Antibacterial activity of pristine PAN and cationic PAN membranes (DABCO- and DMAEMA-based) were evaluated using a modified ASTM E2149−20 assay with S. aureus (ATCC 29213). Membrane samples (1.0 cm²) were incubated with 2 mL of bacterial suspension (10^5 CFU/mL) for 2 h. The suspensions and their serial dilutions (104–10² CFU/mL) were plated on Columbia agar and incubated for 17 h before colony counting. Experiments were performed in triplicate, with pristine PAN (Ctrl) as the control. Colony counts >250 were recorded as too numerous to count.

11) NMR-Spectra

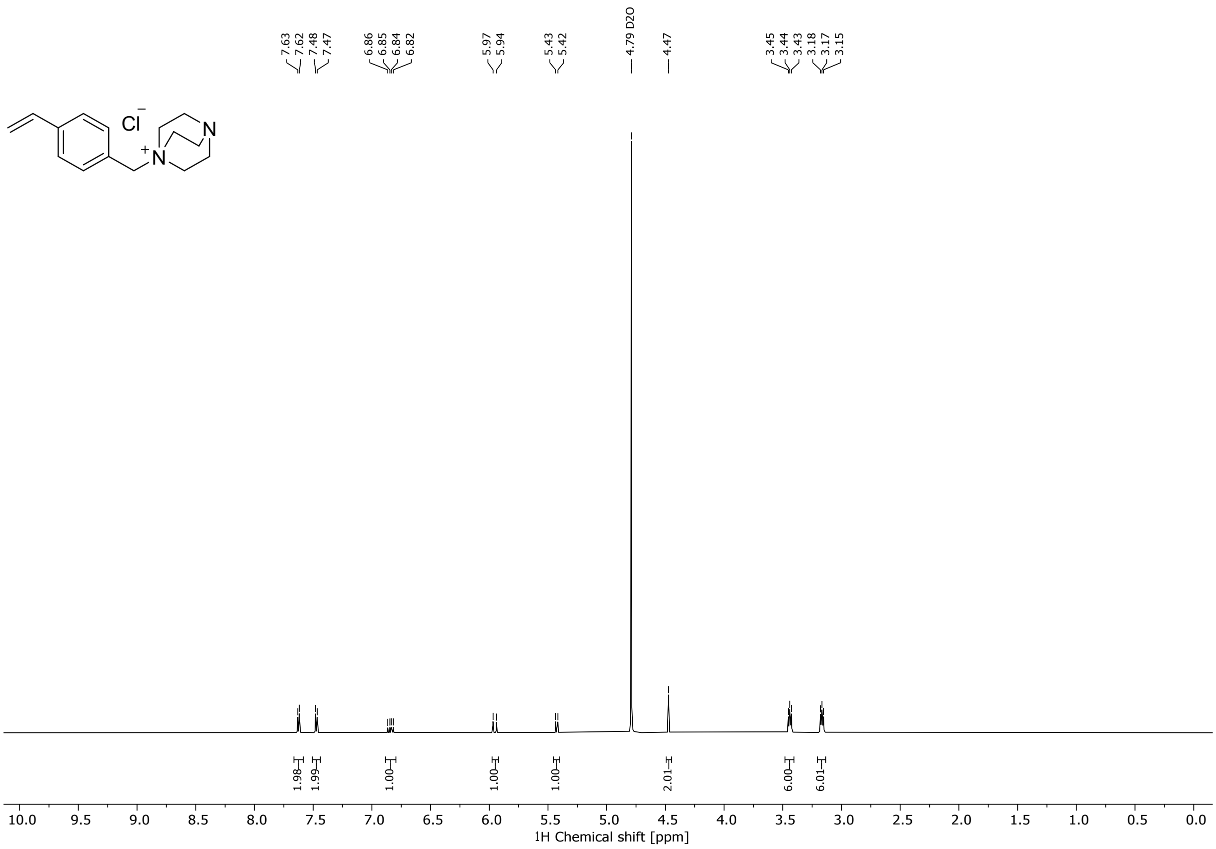

Figure S15: ^{1}H-NMR spectrum (600 MHz, D_2O) of VBD.

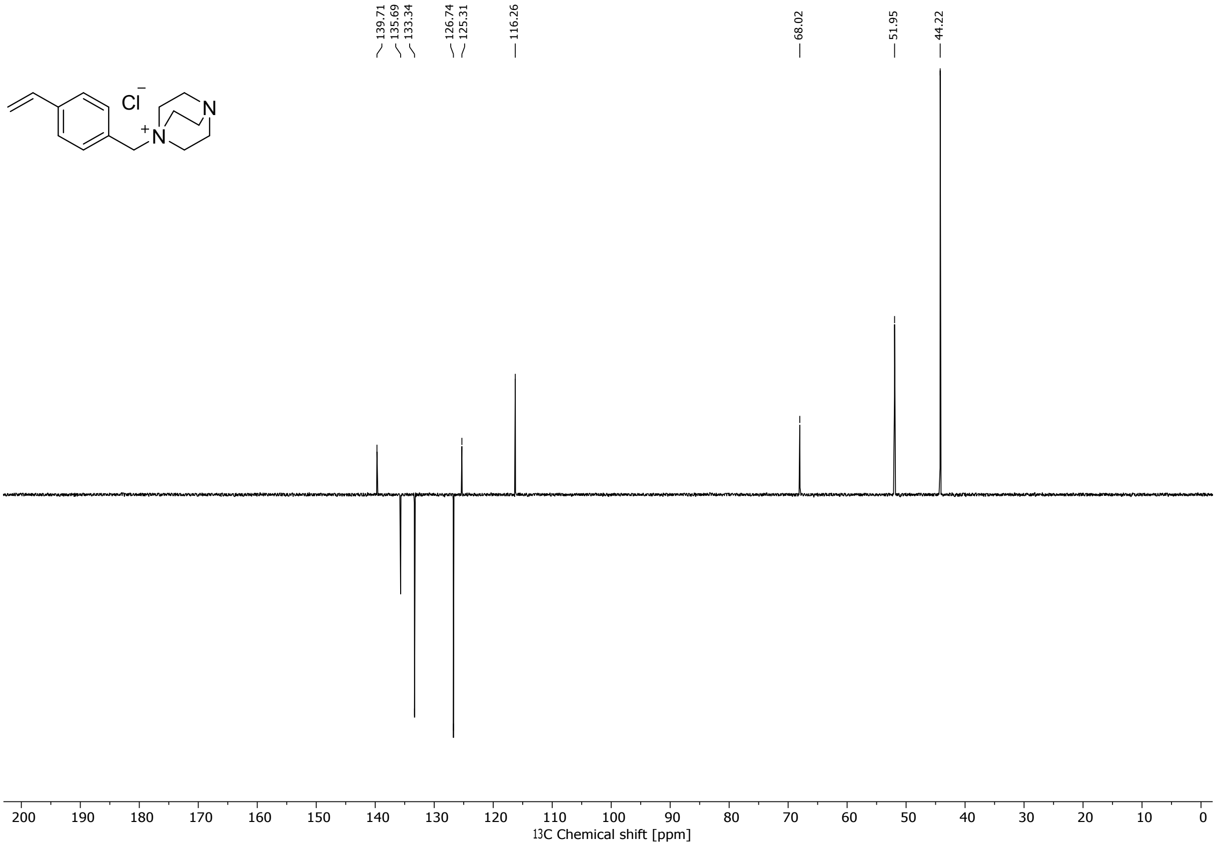

Figure S16: ^{13}C-NMR-DEPTQ spectrum (150 MHz, D_2O) of VBD.

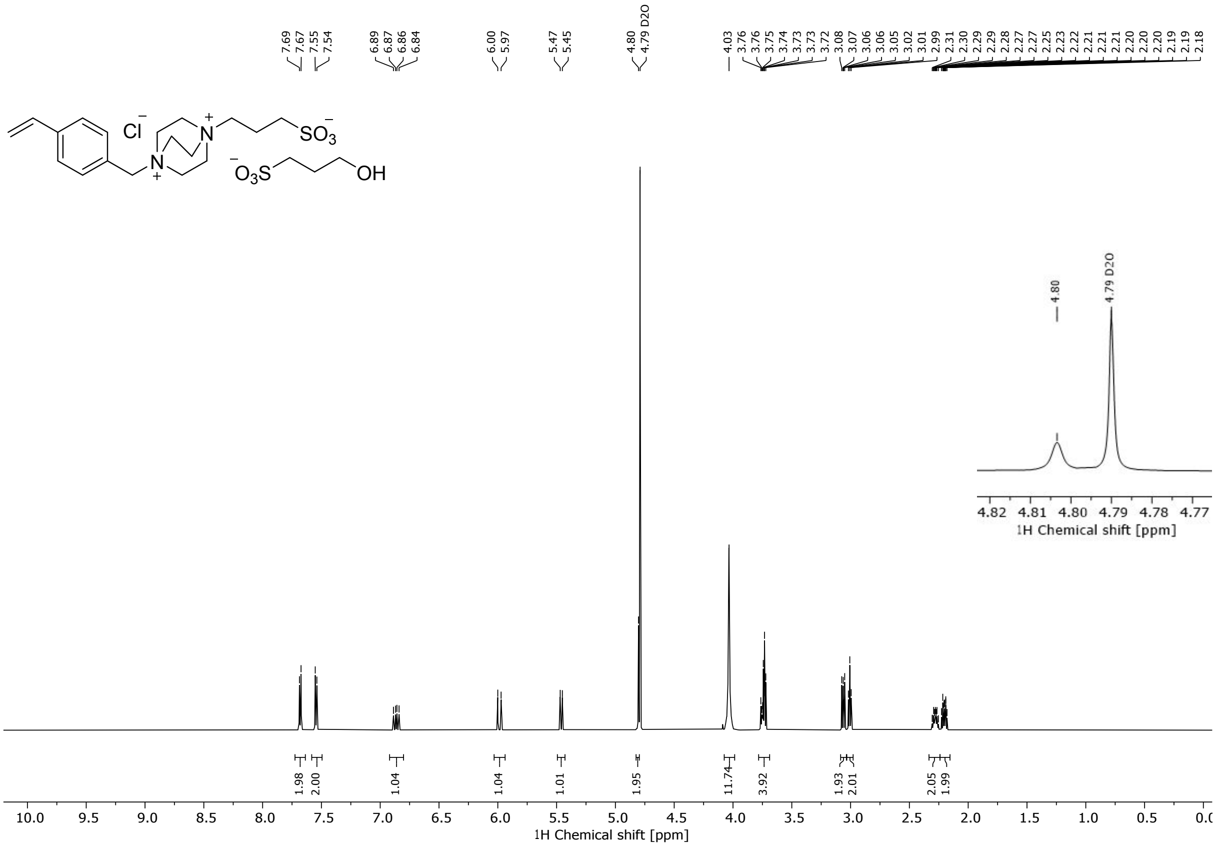

Figure S17: ^{1}H-NMR spectrum (600 MHz, D_2O) of VBD-SB.

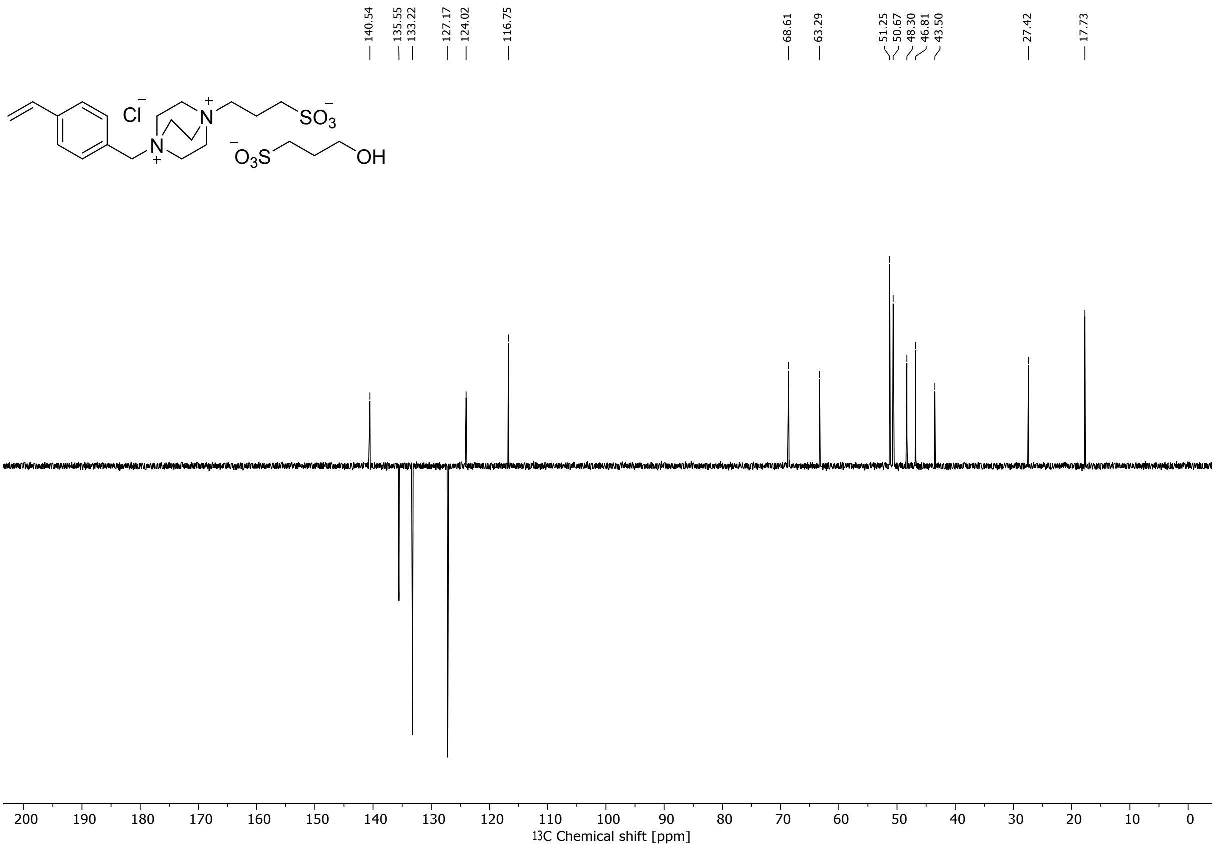

Figure S18: ^{13}C-NMR-DEPTQ spectrum (150 MHz, D_2O) of VBD-SB.

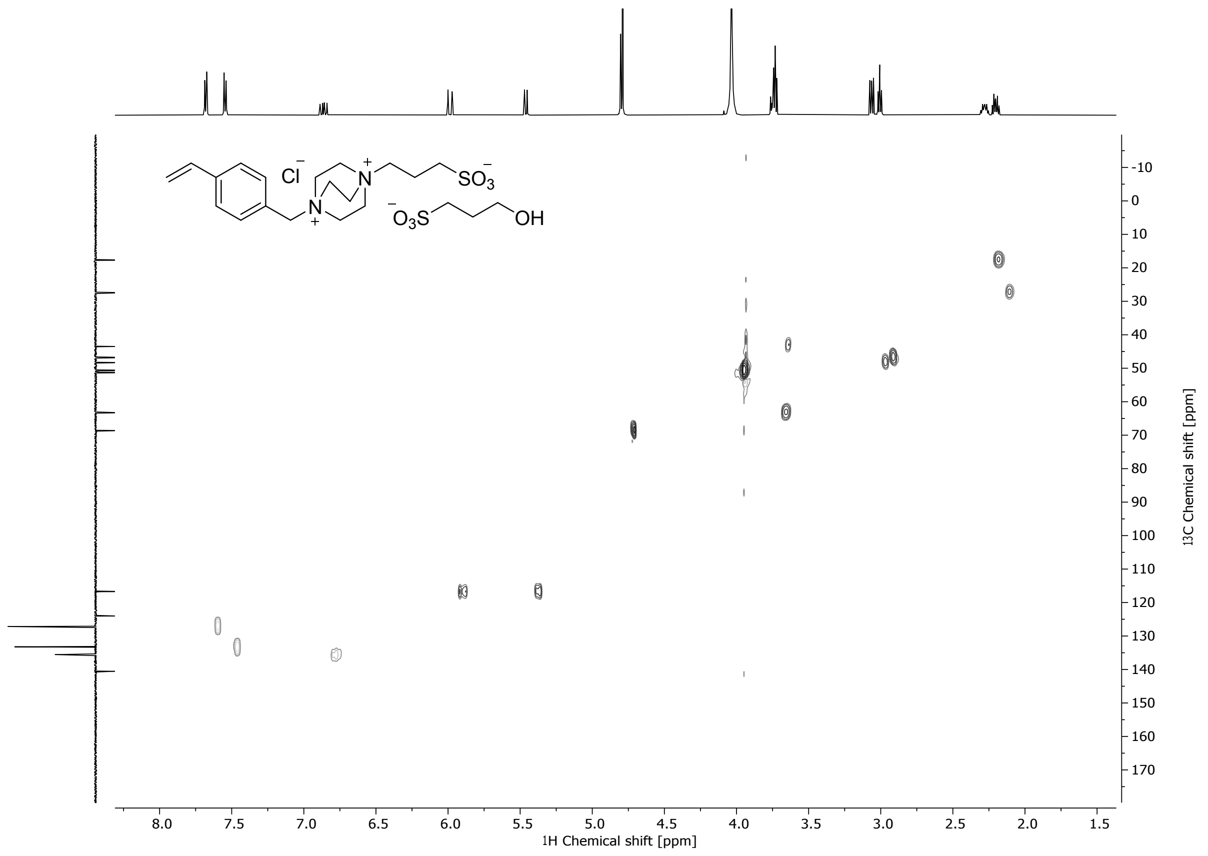

Figure S19: HSQC 2D-NMR spectrum (500 MHz, 150 MHz, D_2O) of VBD-SB.

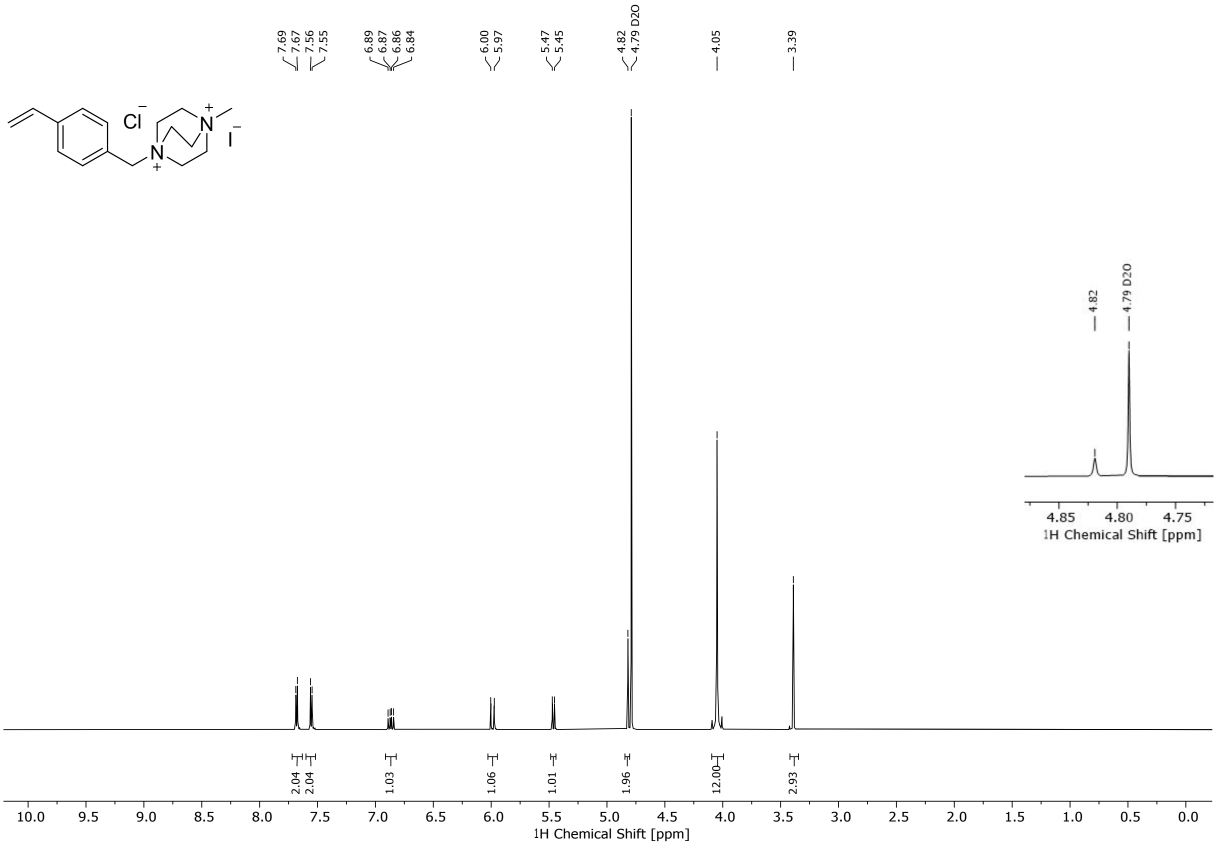

Figure S20: ^{1}H-NMR spectrum (600 MHz, D_2O) of VBD-ME.

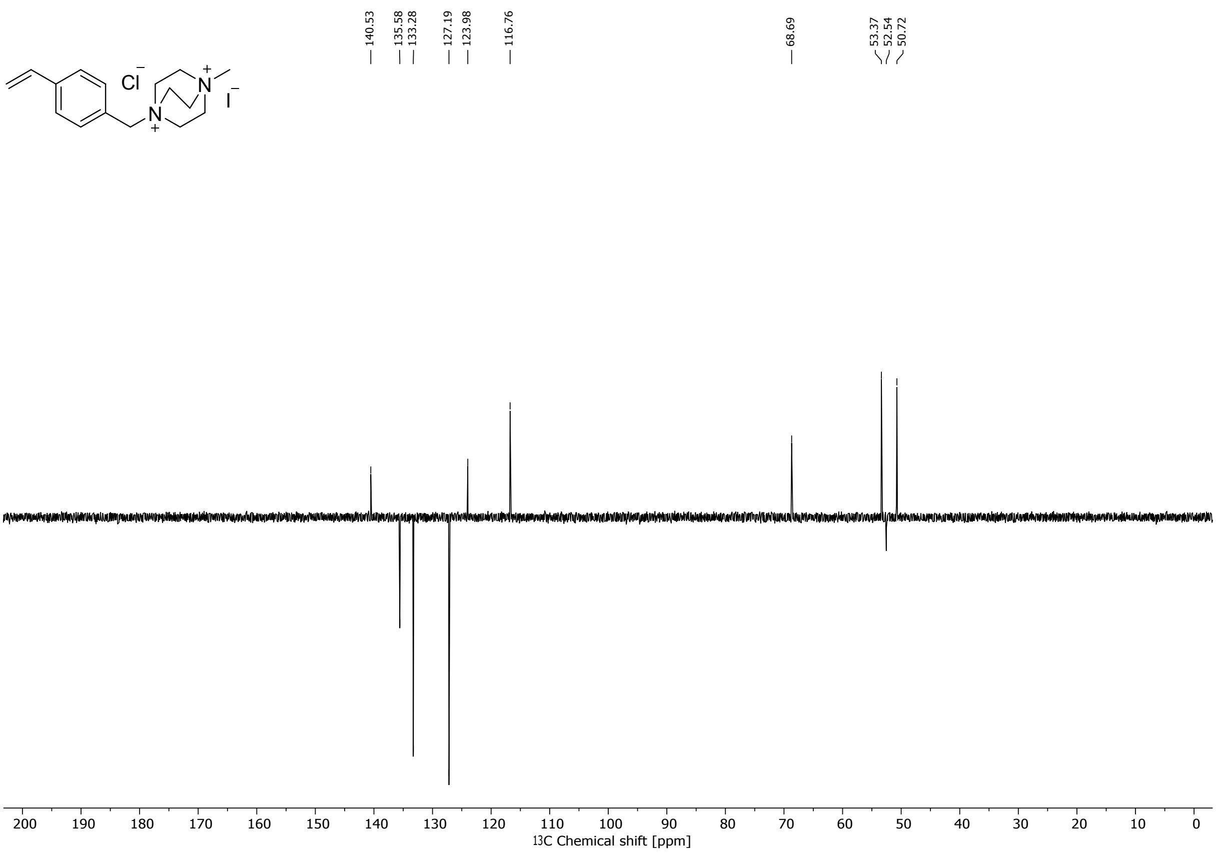

Figure S21: ^{13}C-NMR-DEPTQ spectrum (150 MHz, D_2O) of VBD-ME.

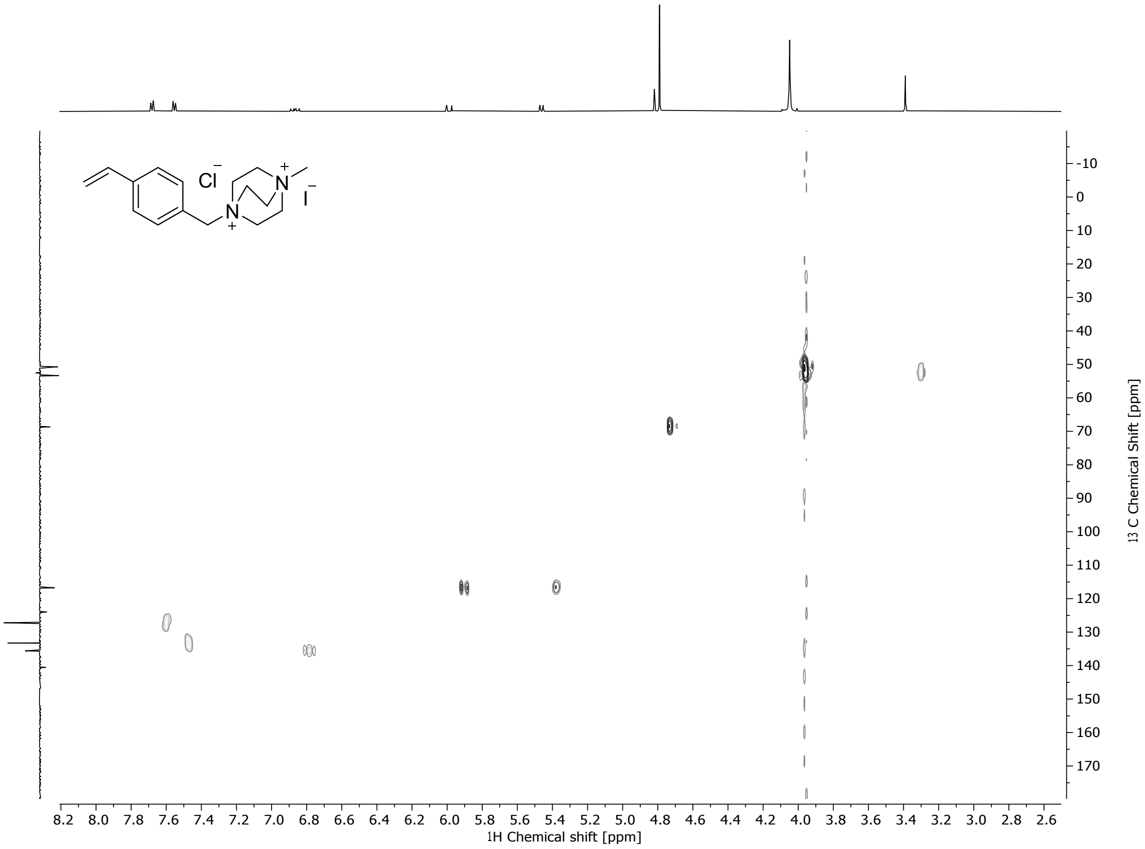

Figure S22: HSQC 2D-NMR spectrum (500 MHz, 150MHz, D_2O) of VBD-ME.

PAN-g-DMAEMA-SB showed a positive zeta potential. This behavior deviates from literature reports, which indicate that sulfobetaine groups generally display negative zeta potential across all pH values.[4] A possible explanation for this discrepancy is the elimination of the sulfobetaine structure, leading to the formation of vinylsulfonate and a positively charged ammonium group. To test this hypothesis, a homopolymer of DMAEMA was synthesized and reacted with 2-bromoethanesulfonate. The reaction conditions were the same as for post-functionalization.

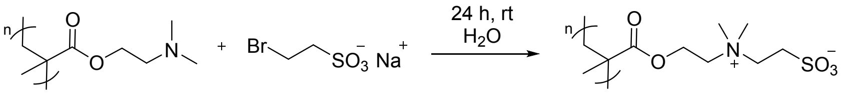

NMR analysis confirmed the elimination reaction, resulting in the formation of vinylsulfonate (Figure S23).

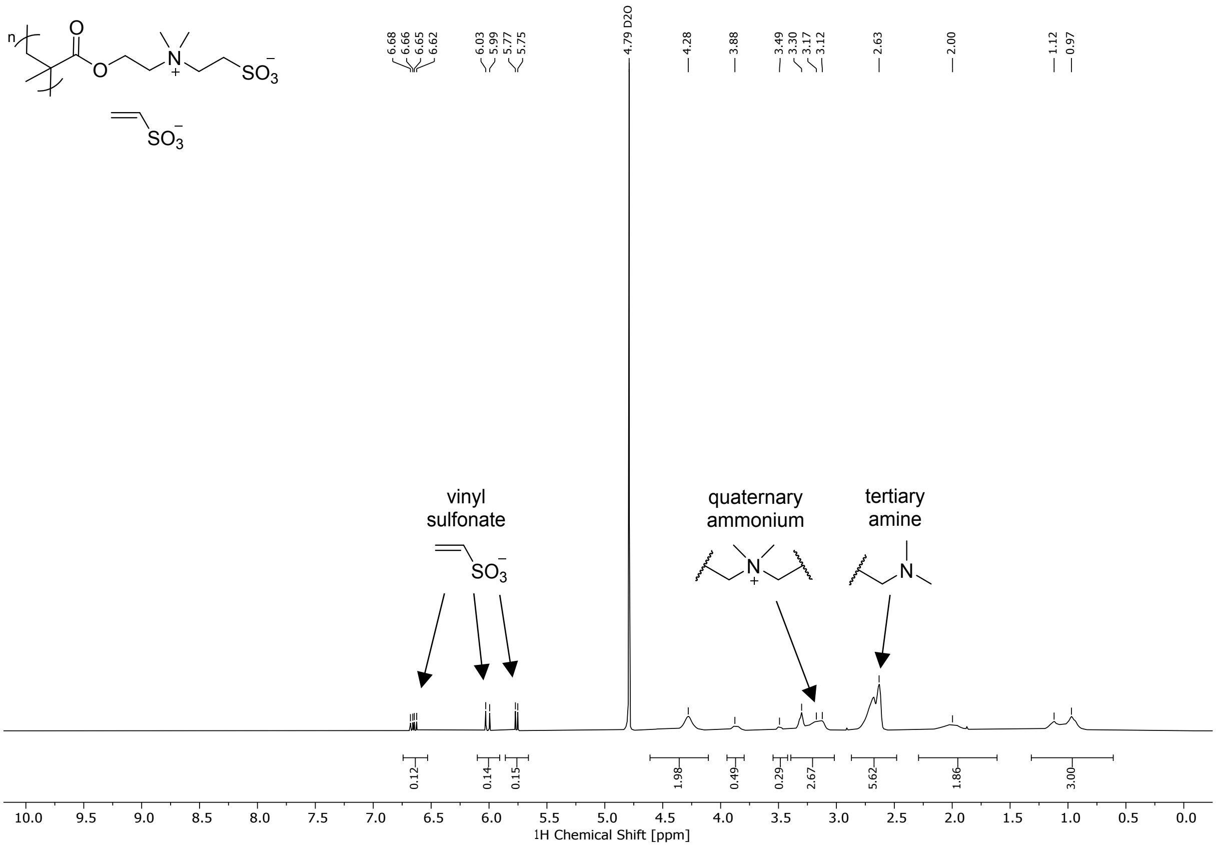

Figure S23: ^{1}H-NMR spectrum (400 MHz, D_2O) of poly(2-((2(methacryloyloxy)ethyl)dimethylammonio)ethane-1-sulfonate) vinyl sulfonate.

The vinylsulfonate could also have been formed by the elimination of 2-bromoethanesulfonate in aqueous solution at elevated temperature. Carboxybetaines with a C2 spacer have been reported to be unstable, existing in equilibrium with the ammonium acrylate salt formed through elimination.[5] In literature, this was not observed for C2 sulfobetaines. Further investigations of other post-modifications revealed no similar elimination products. In summary, it can be concluded that the surface was not completely functionalized. It consists of a mixture of sulfobetaines, protonated amines, and non-protonated tertiary amines. When the tertiary amines are protonated, the surface carries an overall positive charge, which is also reflected in the zeta potential data.

12) HRMS-Spectra:

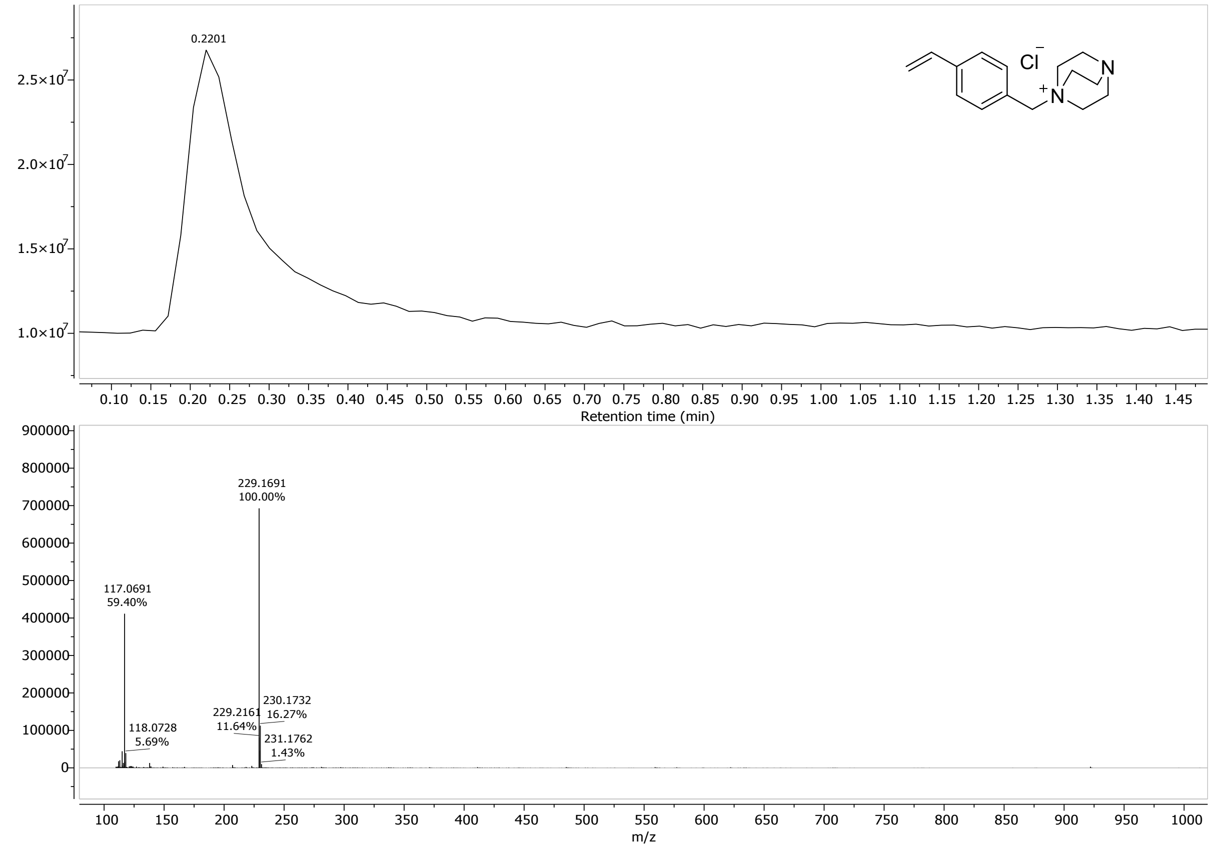

Figure S24: ESI(+)-HRMS: Chromatogram and mass spectrum of VBD.

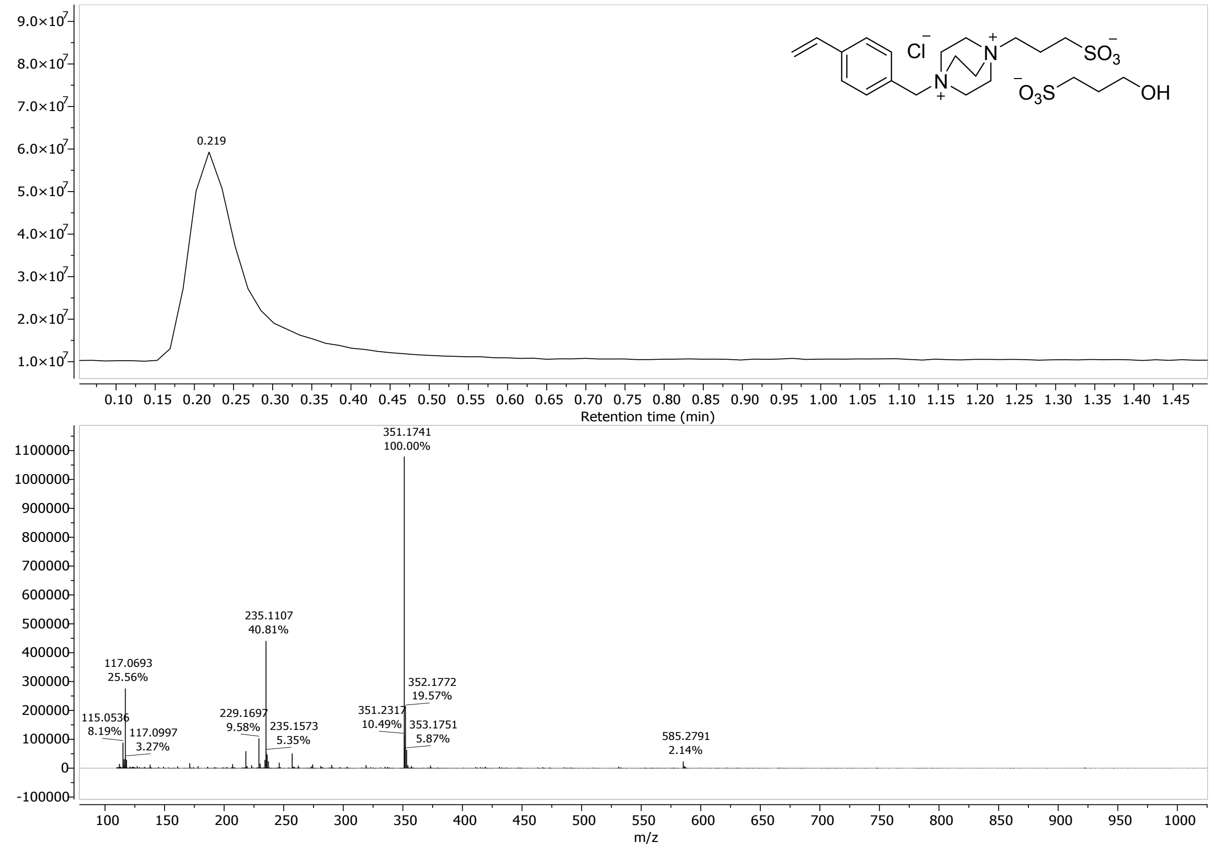

Figure S25: ESI(+)-HRMS: Chromatogram and mass spectrum of VBD-SB.

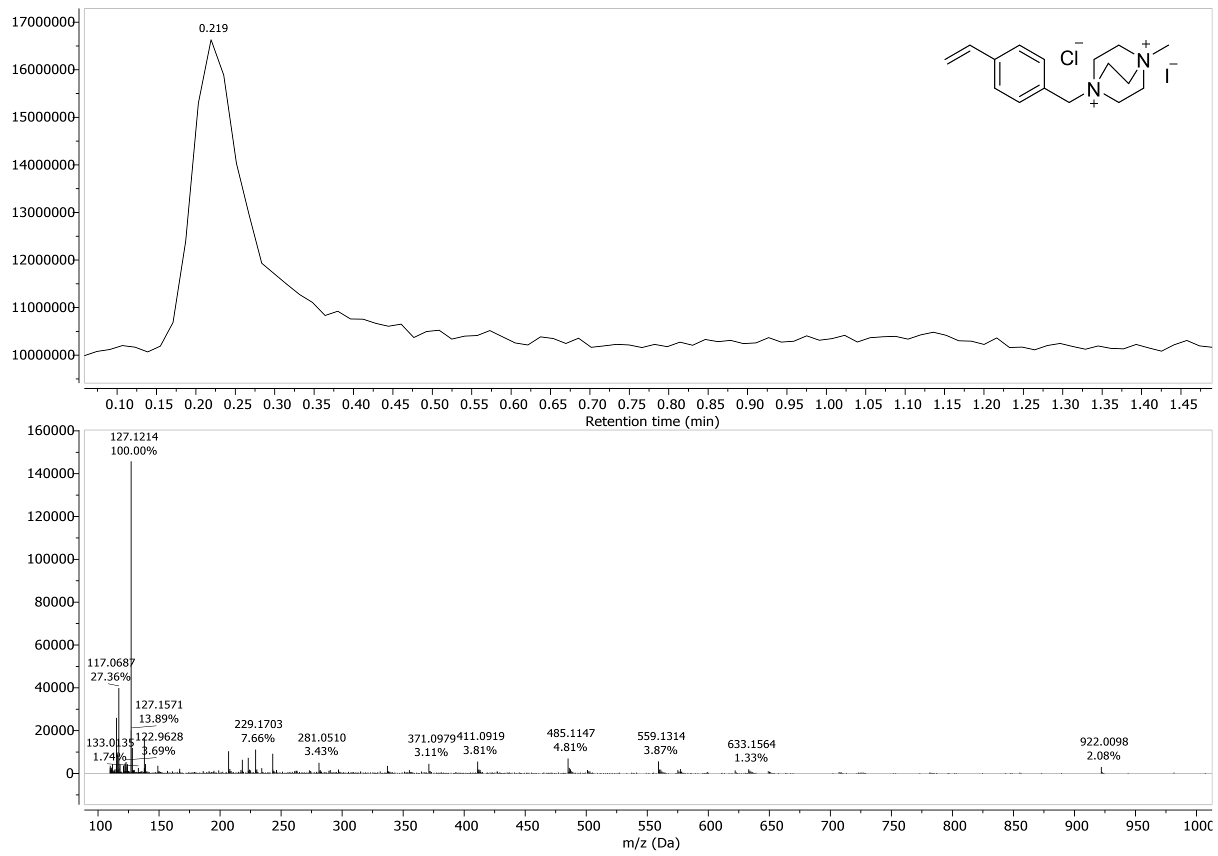

Figure S26: ESI(+)-HRMS: Chromatogram and mass spectrum of VBD-ME.

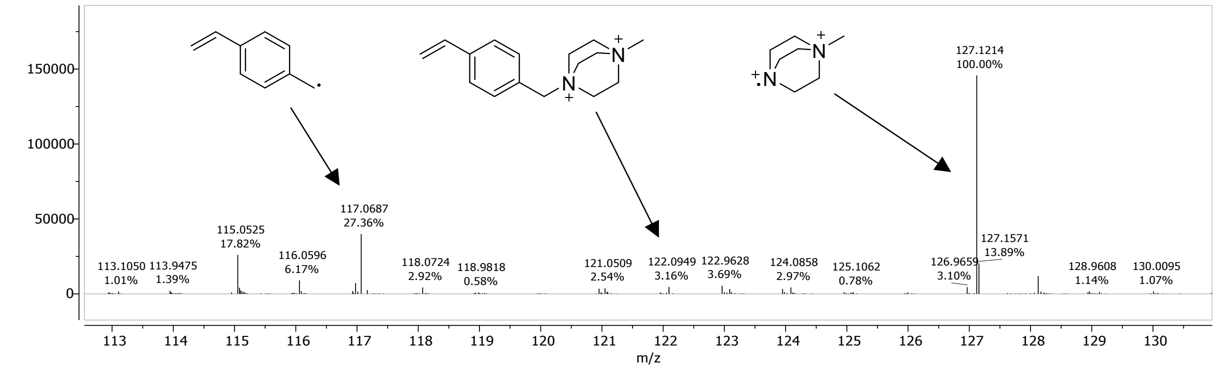

Figure S27: ESI(+)-HRMS: Zoomed-in mass spectrum of VBD-ME.

13) Literature

(1) Burmeister, N.; Zorn, E.; Preuss, L.; Timm, D.; Scharnagl, N.; Rohnke, M.; Wicha, S. G.; Streit, W. R.; Maison, W. Low-Fouling and Antibacterial Polymer Brushes via Surface-Initiated Polymerization of a Mixed Zwitterionic and Cationic Monomer. *Langmuir* **2023**, *39* (49), 17959-17971. DOI: 10.1021/acs.langmuir.3c02657.

(2) Decker, C.; Zahouily, K.; Decker, D.; Nguyen, T.; Viet, T. Performance analysis of acylphosphine oxides in photoinitiated polymerization. *Polymer* **2001**, *42* (18), 7551-7560. DOI: 10.1016/s0032-3861(01)00221-x.

(3) Green, W. A. Industrial photoinitiators. A technical guide. *CRC Press* **2010**, (1), 17-46. DOI: https://doi.org/10.1201/9781439827468.

(4) Guo, S.; Jańczewski, D.; Zhu, X.; Quintana, R.; He, T.; Neoh, K. G. Surface charge control for zwitterionic polymer brushes: Tailoring surface properties to antifouling applications. *Journal of Colloid and Interface Science* **2015**, *452*, 43-53. DOI: https://doi.org/10.1016/j.jcis.2015.04.013.

(5) Laschewsky, A. Structures and Synthesis of Zwitterionic Polymers. *Polymers (Basel)* **2014**, *6* (5), 1544-1601. DOI: 10.3390/polym6051544.
